# A Comprehensive Genome-Wide Map of Autonomously Replicating Sequences in a Naive Genome

**DOI:** 10.1371/journal.pgen.1000946

**Published:** 2010-05-13

**Authors:** Ivan Liachko, Anand Bhaskar, Chanmi Lee, Shau Chee Claire Chung, Bik-Kwoon Tye, Uri Keich

**Affiliations:** 1 Department of Molecular Biology and Genetics, Cornell University, Ithaca, New York, United States of America; 2 Department of Computer Science, Cornell University, Ithaca, New York, United States of America; 3 School of Mathematics and Statistics F07, University of Sydney, Sydney, Australia; UMDNJ-New Jersey Medical School, United States of America

## Abstract

Eukaryotic chromosomes initiate DNA synthesis from multiple replication origins. The machinery that initiates DNA synthesis is highly conserved, but the sites where the replication initiation proteins bind have diverged significantly. Functional comparative genomics is an obvious approach to study the evolution of replication origins. However, to date, the *Saccharomyces cerevisiae* replication origin map is the only genome map available. Using an iterative approach that combines computational prediction and functional validation, we have generated a high-resolution genome-wide map of DNA replication origins in *Kluyveromyces lactis*. Unlike other yeasts or metazoans, *K. lactis* autonomously replicating sequences (*Kl*ARSs) contain a 50 bp consensus motif suggestive of a dimeric structure. This motif is necessary and largely sufficient for initiation and was used to dependably identify 145 of the up to 156 non-repetitive intergenic ARSs projected for the *K. lactis* genome. Though similar in genome sizes, *K. lactis* has half as many ARSs as its distant relative *S. cerevisiae*. Comparative genomic analysis shows that ARSs in *K. lactis* and *S. cerevisiae* preferentially localize to non-syntenic intergenic regions, linking ARSs with loci of accelerated evolutionary change.

## Author Summary

DNA replication is an evolutionarily conserved, cell cycle–regulated, spatially and temporally coordinated mechanism in eukaryotes. It is initiated by the binding of the Origin Recognition Complex (ORC) to multiple replication origins. While the ORC is highly conserved, its DNA binding specificity and the primary sequences of replication origins are not. Comparative functional genomics is an obvious approach to addressing questions about the positional conservation and chromosomal determinants of replication origins. However, to date, *Saccharomyces cerevisiae* is the only eukaryote with a complete genome-wide replication origin map, one which took three decades to compile. We have devised an iterative approach, combining computational prediction and functional validation by direct cloning of replication origins that efficiently identifies a high resolution, near complete repertoire of replication origins in *Kluyveromyces lactis.* Comparing these two yeast genome maps provides a wealth of information about the DNA elements and positional conservation of replication origins in these two distantly related yeast species. This approach is generally applicable to the construction of high-resolution genome maps of evolutionarily conserved sequences associated with assayable biological functions. Rapid generation of comprehensive functional maps of uncharacterized genomes is critical to whole genome studies of all biological functions.

## Introduction

The first step in the initiation of DNA synthesis in eukaryotes is the binding of the Origin Recognition Complex (ORC) to the replication origin [1]. However, replication origins have very different characteristics depending on the organism and sometimes the locale within the genome of an organism [2], [3]. In the budding yeast *Saccharomyces cerevisiae*, replication origins are short, modular, *cis-*acting sequences that support the autonomous replication of extrachromosomal plasmids [4]–[6]. The *S. cerevisiae* ORC binds to a T-rich 17 bp motif termed the ARS Consensus Sequence (ACS) that is sometimes supported by interaction with nearby B-elements [1], [7]. In the fission yeast *Schizosaccharomyces pombe*, replication origins are degenerate A/T-rich sequences which ORC binds in a stochastic manner [8]–[11]. Intergenic sequences 500 bp in length that contain >75% A-T are potential initiation sites for DNA replication. A genome-wide map based on high resolution tiling microarray indicates that there are 401 strong origins and 503 putative weak origins spaced at an average length of ∼14 kb in clusters throughout the *S. pombe* genome [12]–[14]. In this respect, the genomic landscape for replication initiation in *S. pombe* resembles that of insect and amphibian embryonic cells [15], [16]. In mammals, some replication origins consist of physically dispersed combinatorial *cis* elements that serve as defined ORC binding sites while others are degenerate sequences that ORC recognizes stochastically [17]. Because of the defined nature of its replication origins, *S. cerevisiae* is the only eukaryote whose entire repertoire of replication origins have been identified, but not without extensive efforts [5], [14], [18]–[23] that took almost three decades. As it is, the *S. cerevisiae* replication origins genome map is less than 100% complete as new ARSs are still being identified with each new study (this study and [24]). Recent work has also made strides towards constructing a genome map of replication origins in another pre-whole-genome duplication (pre-WGD) budding yeast *Lachanacea kluyveri* [25]. However, the great divergence between these pre-WGD species inhibits meaningful comparative genomic analyses. Studies of additional yeast species that bridge these wide divides would greatly enhance informative evolutionary comparisons.

In this study, we have devised an approach that combines computational prediction and experimental validation to study the genome-wide location of replication origins of *Kluyveromyces lactis.* The motivation for this study is to investigate the evolutionary changes that lead to the divergence of replication origins in eukaryotes. To facilitate our goal, we developed new tools to map and study replication origins at a genome-wide scale with greater efficacy. We chose *K. lactis* as our model because it is distant from *S. cerevisiae* – they diverged prior to WGD in the evolution of yeast species about 100 million years ago [26], [27], and its emergence is dated between that of *S. pombe* and *L. kluyveri* in the fungal tree of life. Another advantage of working with this species is that previous studies of three *K. lactis* autonomously replicating sequences (ARSs) showed that they are defined sequences, but with features that are significantly different from *S. cerevisiae* and *S. pombe* ARSs [28]–[30]. These factors make the *K. lactis* genome an excellent model for our origin mapping study since it encompasses both similarities and differences with other established models. This study focuses on further characterizing the DNA replication initiation sites of *K. lactis* at a genomic scale.

## Results

### Isolating *K. lactis* ARSs

**Figure 1:**
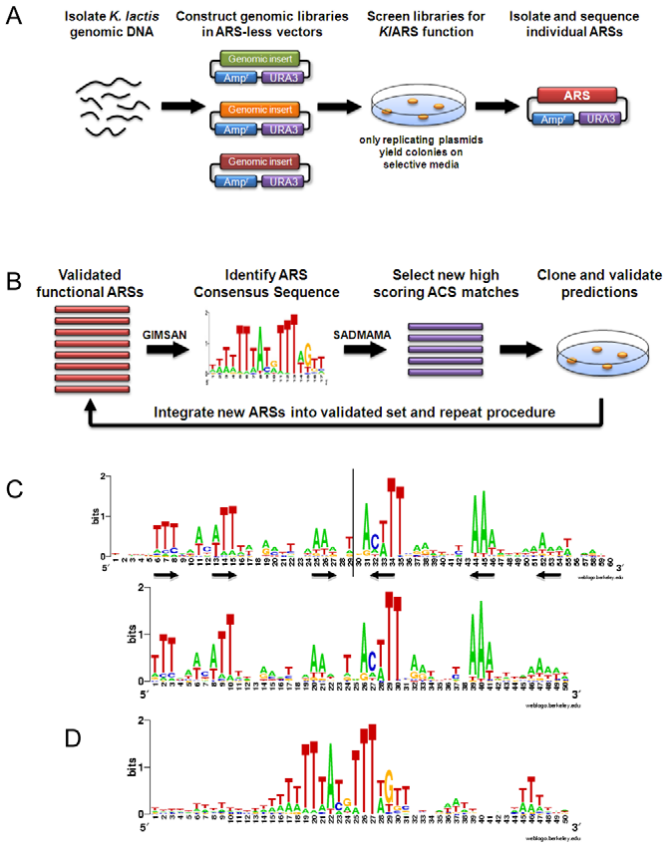
Isolation and characterization of *K. lactis* ARSs. (A) Schematic of the ARS screen in *K. lactis*. (B) Schematic for the iterative predict-and-verify approach for identifying genomic ARSs. (C) Logos of *Kl*ACS [66]. Top panel, *Kl*ACS based on the initial 69 *Kl*ARSs. Bottom panel, *Kl*ACS based on 148 verified *Kl*ARSs. Arrows indicate an inverted repeat motif centered about an axis of symmetry marked by a vertical line. (D) An extended 50 bp motif of the *S. cerevisiae* ACS motif is shown in comparison with the *Kl*ACS.

We began our study of the genome-wide locations of *K. lactis* ARSs by constructing genomic libraries of *K. lactis* in a non-replicating *URA3* vector and screening these libraries for autonomous replication function in a *uraA* (the *K. lactis* equivalent of *ura3*) auxotrophic strain of *K. lactis* [31] (Figure 1A). Efficient plasmid replication was required for colony growth on medium lacking uracil, therefore only ARS bearing plasmids yielded colonies. The plasmids were isolated from yeast colonies, sequenced, and used to re-transform yeast to confirm ARS function. Sixty-nine unique ARS-containing fragments were identified from this non-saturating screen.

Due to the random nature of the screen, the ratio of novel to rediscovered ARSs rapidly declined after several rounds of screening. To overcome the diminishing returns of our screen we adopted a more targeted, iterative, approach to identify additional ARSs. Each cycle of the procedure consisted of compiling a list of ∼20 new candidate ARSs whose functionality would then be manually verified (Figure 1B). In a manner somewhat reminiscent of PSI-BLAST [32], the prediction step of each cycle was made based on the information gained from all previous cycles including the initial set of 69 *K. lactis* screened ARSs. Specifically, applying the *de-novo* motif finder GIMSAN [33] to the set of all hitherto verified *K. lactis* ARSs, we defined a new ACS Position Weight Matrix (PWM). Visual inspection of the *Kl*ACS motif found by GIMSAN applied to the initial set of screened *Kl*ARSs strongly suggested that the correct motif length was 50 bp (Figure 1C, top panel). Importantly, this agreed with the predicted length from previous work on two genomic *Kl*ARSs [29], [30]. We therefore consistently instructed GIMSAN to search for a motif of length 50 bp in all the iterations. The newly GIMSAN-defined ACS PWM was then used by the SADMAMA application [34] to scan the *K. lactis* genome for new high scoring matches. Due to the discrete nature of SADMAMA's score, in some cases, the new list of top candidates could either have slightly less or slightly more than 20 candidates. We consistently chose the latter. Each batch of the approximately 20 best matches were then used to identify candidate ARSs by cloning a genomic fragment encompassing the ACS and 200 bp of flanking DNA from both sides of the predicted sequence into the non-replicating vector and assaying for ARS function (Figure 1B).

**Figure 2:**
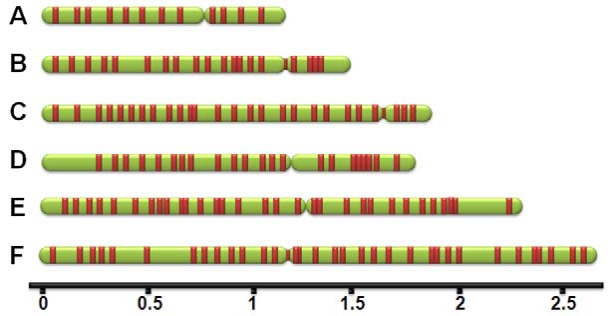
A representation of genomic locations of *Kl*ARSs. The 6 *K. lactis* chromosomes are shown in green with red blocks representing the *Kl*ARSs (*Kl*ARS size not to scale). The scale represents Megabases of genomic DNA.

Our initial screen indicated that, as in *S. cerevisiae*, the vast majority (66 out of 69) of *K. lactis* ARSs resided in intergenic regions. We therefore largely restricted our predictions to the cases where the predicted ACS falls in *K. lactis* intergenic regions. Starting from the set of 69 initially screened ARSs in 5 predict-and-verify cycles we define a total of 123 candidate intergenic *K. lactis* ARSs, (Dataset S1). Of these, five candidates resisted multiple attempts at PCR and could not be verified, 75 were verified to have *K. lactis* ARS functionality, whereas 43 turned out to be non-functional. In addition, using a combination of other techniques to computationally predict ARSs (see Materials and Methods), we verified another 4 *Kl*ARSs at a considerably higher cost of 32 negatively verified sequences (Dataset S2). Thus, we verify a total of 148 *K. lactis* ARSs (69 from our original screen and 79 verified predictions), 145 of which lie in annotated intergenic regions (Figure 2, Table S1).

How complete is our coverage of *K. lactis* ARSs, or, how many ARSs does *K. lactis* have? To address these two virtually equivalent questions more effectively, we set the following conditions. Due to the paucity of ARSs in coding regions, which account for 70% of the genome, we limited our estimation to the number of ARSs in intergenic regions of *K. lactis* as in doing so we minimized the variability in our estimate. In addition, due to the repetitive structure of the ribosomal DNA, telomeric and sub-telomeric regions, we only considered unique representative ARSs in these regions.

### Estimating the complete repertoire of *K. lactis* ARSs

We estimated the total number of non-repetitive intergenic ARSs in the *K. lactis* genome using a technique known as “leave-one-out” (LOO) to account for the missing or unseen ARSs. One of the original 69 screened ARSs was systematically left out from the existing sample as the new training set to test how well the specific PWM predicts the ACS locations in the held out data [35]. Using this general approach we devised a new method to find a confidence interval for the number of intergenic *Kl*ARSs and conclude that [145, 156] is the 95% confidence interval for the number of non-repetitive intergenic *Kl*ARSs [36].

Briefly, the process by which we estimate the total number of ARSs is independent of the predict-and-verify process used to actually find the ARSs. This estimation is based only on PWMs derived from the original set of screened ARSs and hence it is not biased by any computational predictions. To estimate the size of a finite set of binding sites **S**, we need a well-defined binding site (such as the *K. lactis* ACS), an initial **random** sample of a subset of binding sites from **S** (which in our case is the initial set of screened ARSs) and a verification procedure that validates a site if and only if it belongs to our set **S** (a *K. lactis* ARS in our example). To verify that our PWM model does not oversimplify the real intricacy of the presumed ORC binding site we tested our estimation procedure on *S. cerevisiae* [36]. We showed that starting from a randomly sampled subset of the set **S** of confirmed *S. cerevisiae* ARSs our 95% confidence interval for the total number of confirmed *S. cerevisiae* ARSs underestimates the true number less than 5% of the time. This result is exactly what one would expect from a 95% confidence interval and it demonstrates that our model (as described in details in [36]) correctly captures the nature of the binding sites.

The estimate of [145, 156] interval for the total set of *K. lactis* ARSs means that we are 95% confident that our coverage of *K. lactis* ARSs is essentially exhaustive. The credibility of this claim is further strengthened by noting that the 16 lowest scored candidates in our 5^th^ and last iterative list include only one verified ARS compared with 13 candidates that were verified to be non-functional while another 2 could not be cloned (Dataset S1).

### Comparison with *S. cerevisiae* ARSs

From the 148 ARSs we verified, a refined 50 bp ACS was derived (Figure 1C, bottom panel and Dataset S3). Visually, there is no significant difference between the initial (top) and refined (bottom) PWM logos in Figure 1C. However, there is a significant difference in terms of the predictive power of the underlying numerical PWMs. Specifically, the top 100 intergenic ACS matches to these two matrices have a negative (non-functional ARS) to positive (functional ARS) ratio of 0.49 for the initial PWM but only 0.28 for the refined PWM. The most notable difference between the *Kl*ACS and the *Sc*ACS is the length (compare Figure 1C and 1D). While the “canonical” *Sc*ACS consists of a 17 bp asymmetric A-T sequence (Figure 1D, nucleotides 15–31) [23], the *Kl*ACS is 50bp in length with short patches of A or T in positions suggestive of an inverted dimeric motif with similar spacing of T patches on each half (Figure 1C, bottom panel).

As expected, *Kl*ARSs have an overwhelming preference for intergenic regions. Only three of the 69 *Kl*ARSs isolated in the initial screen are located in coding sequences and one of these three also happens to be in a repetitive telomeric region with conflicting annotations. Moreover, all of the 11 high scoring candidates we verified outside of intergenic regions (not part of our predict-and-verify procedure) turned out to be non-functional.

**Figure 3:**
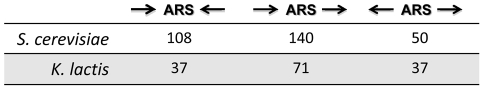
Orientation of transcripts flanking *S. cerevisiae* and *K. lactis* ARSs. ARSs from *S. cerevisiae* and *K. lactis* genomes are categorized with respect to flanking transcription. The headings display the transcription direction from the genes flanking the ARS.

Nieduszynski *et al*. [37] observed that *Sc*ARSs show a clear bias for intergenes between convergently transcribed over intergenes between divergently transcribed genes. Indeed, among the 296 verified *Sc*ARSs for which we could find flanking pairs of genes (f = forward, r = reverse) we found 108 fr (both genes transcribing toward ARS), 50 rf (both genes transcribing away from ARS), 140 ff + rr (one gene transcribing towards ARS), or more than a 2∶1 ratio between converging and diverging flanking gene pairs (Figure 3). This distinct bias completely disappears in *Kl*ARSs where the strand statistics of flanking genes are: 37 (fr), 37 (rf), 71 (ff + rr).

Assuming that the total number of *Kl*ARSs is equal to the projected upper bound of 156, the average spacing of ARSs in *K. lactis* is 69 kb compared to the 37 kb estimated for *S. cerevisiae* based on the total number of 323 verified *Sc*ARSs [37]. Although a similar estimate for the number of ARSs has not been carried out for *S. cerevisiae*, in testing our predict-and-verify approach we were able to isolate 3 new *Sc*ARSs in this study (see Text S1). The composite genome map and coordinates of the 148 *Kl*ARSs and predicted ACSs are shown in Figure 2 and Table S1.

### The 50bp ACS is necessary and largely sufficient for ARS function

**Figure 4:**
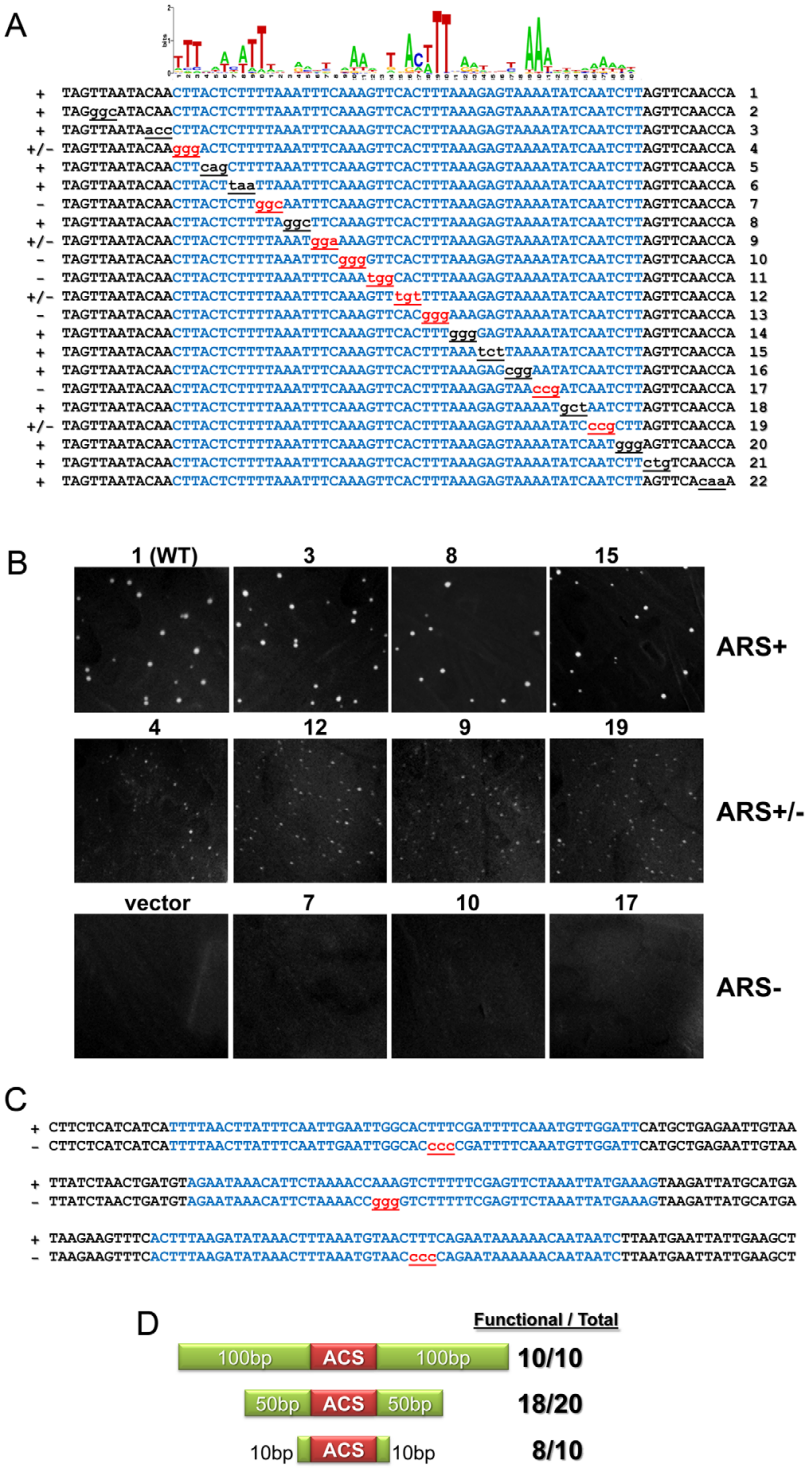
*Kl*ACS is necessary and largely sufficient for *Kl*ARS function. (A) Site-directed mutagenesis was used to replace trinucleotides in *Kl*ARS515. The mutant plasmids were tested for ARS activity. The mutated bases are underlined, and ARS function is indicated on the left and by the text color of the mutated nucleotides. The predicted *Kl*ACS motif is highlighted in blue. Mutants that did not affect function (+) are highlighted in black, while mutants affecting ARS function (+/− and −) are colored in red. Mutants that completely destroyed ARS function are indicated as (−), mutants that support the growth of minute colonies but contain dead cells on restreaking are indicated as (+/−) and mutants that had no effect are indicated as (+). Numbers on the right of the sequences are shown for reference. (B) Pictures of *K. lactis* transformed with mutant ARS plasmids on selective medium plates after 6 days of growth at 30°C. Plasmids with ARS activity comparable to the wild type plasmid (ARS+) are shown on the top row. Plasmids with weak ARS activity (ARS+/−) are shown in the middle row. Plasmids which show no visible ARS activity (ARS-) are on the bottom row. Numbers above the pictures correspond to mutant constructs from Figure 4A. Multiple clones of all plasmids were tested. Representative plasmids are shown. (C) ARS sequences of *Kl*ARS618, *Kl*ARS612 and *Kl*ARS524 were mutagenized to test the necessity of the predicted *Kl*ACS motif for ARS function. In all cases (6 total, see Table S1) the predicted mutation destroyed *Kl*ARS function. Each of the full *Kl*ARS sequences is 451bp. (D) DNA flanking the *Kl*ACS motif was truncated and the resulting sequences were tested for ARS function. The number of shortened *Kl*ARSs that retained function is shown on the right and Table S1.

The definition of a defined replication origin, as opposed to stochastic initiation sites, is first, that it is an ARS and second, that it is “mutable” or contains a specific sequence motif that when mutated would destroy ARS activity. To investigate whether the *Kl*ACS is necessary for function, and to define it functionally, we mutated tri-nucleotides in *Kl*ARS515 across the span of the *Kl*ACS and flanking DNA. We constructed and tested a total of 21 mutants (Figure 4A). From this set, 9 mutations either destroyed or weakened *Kl*ARS function (“weakened” ARS mutants produced very small colonies, which did not show growth if re-streaked or inoculated into liquid medium), while 12 mutants did not have a noticeable effect on *Kl*ARS activity (Figure 4B). Nucleotides essential for *Kl*ARS function clustered in the central region of the *Kl*ACS, but mutating nucleotides in the distal regions of the *Kl*ACS also had a strong deleterious effect on *Kl*ARS function. The mutagenesis analysis showed that functionally essential nucleotides were interspersed with non-essential ones, in a pattern corresponding with conserved and non-conserved positions in the PWM that form the putative inverted dimeric motif.

To test whether mutating the *Kl*ACS would destroy function in other *Kl*ARSs, we made trinucleotide substitutions in 5 additional *Kl*ARSs that should destroy the *Kl*ACS motif. In all 5 cases (Figure 4C, only 3 shown), these mutations destroyed the ARS function of the target sequence confirming that the *Kl*ACS motif is necessary for *Kl*ARS function.

To investigate to what extent this motif is sufficient for *Kl*ARS function, we systematically shortened DNA flanking the *Kl*ACS and then tested for ARS function (Figure 4D). We truncated 10 ARSs leaving 100 bp flanking either side of the *Kl*ACS and all 10 retained function (Figure 4D, top row). Further truncating these and 10 others to leave only 50 bp flanks did not destroy ARS function except for two (Figure 4D, middle row, Table S1). From the remaining 18 ARSs, we further truncated 10 to short 10 bp flanks. Eight of these shortest constructs retained ARS function (Figure 4D, bottom row, Table S1). Interestingly, the two non-functional shortest ARS constructs had the lowest and 4th lowest scored *Kl*ACS match among these 10 ARSs. These findings suggest that the *Kl*ACS is largely sufficient for function and that *Kl*ARSs rely almost exclusively on the ACS motif and not on flanking sequences. This motif dependence is profoundly different from *S. cerevisiae*, where the ACS is not sufficient for function and from *S. pombe* and metazoans, where no consensus motif can be identified [3].

### *K. lactis* ARSs are chromosomal replication origins

**Figure 5:**
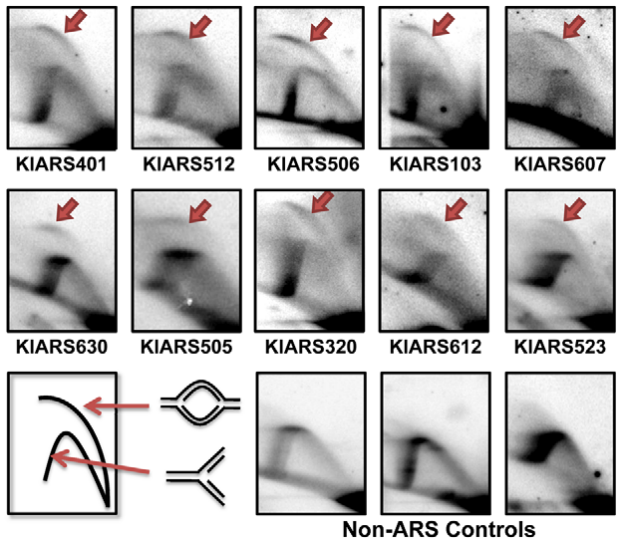
Genomic replication initiation shown by 2D gel electrophoresis. 10 randomly chosen *Kl*ARSs and three non-ARS sequences were analyzed. The name of the ARS tested in its genomic context is indicated below each picture. Red arrows indicate “bubble arcs” caused by replication initiation events at probed loci. All experiments were performed on asynchronous log-phase cultures.

While the ARS assay has been commonly used as a surrogate for replication origin function, it is important to verify that plasmid-borne ARSs are origins of replication in their native genomic loci. Ten randomly chosen *Kl*ARS regions and 3 non-ARS control regions were analyzed for initiation activity by 2-D gel electrophoresis analysis [38] (Figure 5). All 10 ARS-containing regions showed an initiation “bubble” arc indicating that initiation of DNA synthesis occurred within a specific site within the regions analyzed. In contrast, none of the non-ARS controls showed origin firing activity within the probed regions (Figure 5). These results suggest that the *Kl*ARSs are also active replication origins in *K. lactis.*

### Nonrepetitive Genomic ARSs are not Positionally Conserved

Eukaryote genomes contain a much larger repertoire of replication origins than the number that is actually used in every cell division [3]. This apparent redundancy suggests that any one particular ARS should not be under strong selection. Thus if indeed a great redundancy is built into the replication initiation process we should see no significant evolutionary conservation in the locations of individual ARSs. To test this hypothesis, we compared the *K. lactis* ARSs characterized in this study and the cumulative *S. cerevisiae* ARSs analyzed to date. Of the 38 *S. cerevisiae* intergenes that are syntenic (with respect to the adjacent gene pairs) to ARS-containing *K. lactis* intergenes, only 2 (5%) contain a verified *Sc*ARS. Incidentally, one of the only two syntenically conserved ARSs is *KARS101* (*Kl*ARS406) which was previously identified by Fabiani et al. [28] and included in our initial set of 69 *K. lactis* screened ARSs (Table S1). When contrasted with the fact that altogether 51 (2.6%) of the verified *Sc*ARSs fall in the 1992 intergenes syntenic between *S. cerevisiae* and *K. lactis*, there is no statistically significant syntenic conservation of ARSs between these two species (insignificant two-sided hypergeometric p-value of 0.51) (Dataset S4 and Text S1). We therefore examined the conservation of *Kl*ARSs at genetic loci where ARSs are known to perform specific functions in *S. cerevisiae*.

The binding of ORC to ACS at *HMLa* and *HMRa* has been shown to be responsible for the silencing of these loci in *S. cerevisiae*. These silencing sites can also function as ARSs. We examined whether any of our *K. lactis* ARSs resides proximal to the orthologous silent mating type loci of *K. lactis*. The closest ARSs are 6 kb and 20 kb away suggesting such dual-function sites are unlikely to exist in *K. lactis.* An interpretation of this observation is that unlike *S. cerevisiae* [39], [40], ORC binding may not be involved in the repression of the silencing of these cryptic cassettes. *K. lactis* may deploy some other means to repress the silent mating type loci as exemplified by *S. pombe* [41], [42].

Ribosomal DNA repeat units are organized in large tandem blocks in eukaryotes such that complete replication of these large chromosomal segments must rely on internal initiation sites. The initiation sites of DNA replication are localized at the non-transcribed region (NTS) within rDNA repeats in every eukaryote analyzed to date including *S. cerevisiae* [43], *S. pombe* [44], mouse [45] and human [46]. We found no exception in *K. lactis. Kl*ARS418 is located at the NTS of rDNA repeats on chromosome D in synteny with *Sc*ARS1200 (SGD notation) shielded from the traffic of actively transcribing rRNA genes.

### ARSs prefer nonsyntenic intergenic regions

Recently published analyses showed that *S. cerevisiae* ARSs co-localize with putative evolutionary breakpoints [47], [48]. Does this observation apply to *K. lactis* ARSs? We found that ARSs are located preferentially in intergenic regions that are syntenically non-conserved between *S. cerevisiae* and *K. lactis*. We found 34.7% (1992/5736) of *S. cerevisiae* intergenic regions are flanked by genes that are syntenic with *K. lactis*, but only 17.3% (51/294) of intergenic *Sc*ARSs lie in syntenic regions. Using a hypergeometric distribution analysis we conclude that *S. cerevisiae* origins prefer nonsyntenic intergenes relative to *K. lactis* (p = 2.0e−11) and that there is no evolutionary conservation in the location of ARSs.

The analysis above is based on a null model where ARSs are randomly assigned to intergenic regions (see Text S1). There is however a possible flaw with this null model. Namely, it does not take into account the fact that the average length of the intergenic regions that are syntenically conserved is 398 bp while the average length of the intergenic regions that are not syntenically conserved is 655 bp. Adjusting this analysis accordingly gives a more conservative but still significant p-value of 0.003 (Dataset S4). Similarly, only 26% (37/145) of *Kl*ARSs localize to syntenic regions compared with 38% (1988/5165) of all *K. lactis* intergenic regions that are syntenically conserved with *S. cerevisiae*. These statistics suggest a preference for non-syntenic intergenic spaces (p = 0.001; adjusted for intergene length, p = 0.04) (Dataset S4).

One possible explanation for the surprisingly small number of ARSs between syntenically conserved genes is that ARSs, particularly in *S. cerevisiae*, are associated with hot-spots for chromosomal instabilities [47]. tRNAs are also believed to be associated with chromosomal instability [49], [50]. In light of the co-localization of ARSs with non-syntenic intergenic regions it is therefore obvious to ask whether they are also associated with tRNAs. This association was previously addressed by Wyrick *et al.* [19] and by Gordon *et al.* [47]. Both studies affirmed a positive association using essentially the same null model. However, previous work did not take into account the fact that *S. cerevisiae* intergenic regions that include tRNAs are considerably longer than intergenic regions that do not include any tRNA: average lengths of 1709 bp vs. 495 bp. Indeed, our own analysis suggests that using the “random intergenic” model (similar to our first model above as well as to that used by Gordon *et al.*) one finds a very significant association between *S. cerevisiae* tRNAs and ARSs (2-sided p-value of 3e-10 see Text S1). However, after adjusting for the different lengths of regions that do and do not contain a tRNA, we find that this association is statistically insignificant (2-sided p-value of 0.5). While both models have validity, our analysis points out the stark difference between the results of these two plausible models. However, unlike in *S. cerevisiae*, we do find statistically significant co-localization between *K. lactis* ARSs and tRNAs (2-sided p-value of 2e-6) even after adjusting for intergene length differences (The average length of a *K. lactis* intergenic region with/without a tRNA is 1114 bp vs 600 bp, respectively, see Text S1). This suggests that the association between ARSs and tRNAs is statistically stronger in *K. lactis* than in *S. cerevisiae*, however the functional relationship between these two elements is not yet understood.

### Analysis of the power of our predict-and-verify approach and its application to predicting *S. cerevisiae* ARSs

How much does the effectiveness of our iterative approach in discovering *K*. *lactis* ARSs owe to the unusually long 50 bp ACS motif? How well can it predict *S*. *cerevisiae* ARSs where the ACS is significantly shorter? In particular, how does it compare with Oriscan [22], another computational approach to discovering *S. cerevisiae* ARSs? Oriscan relies on a fairly complicated computation model that surveys a much larger region around the ACS. Our method, on the other hand, simply models the ACS, and relies on experimental feedback to help it successively improve the ACS PWM.

To ensure a fair comparison between the two approaches we adopted the same training set of 26 *S. cerevisiae* ARSs that was used in the Oriscan study. We also used the same initial 17 bp PWM that the authors compiled from all experimentally verified ACS matches in this training set. We next applied our iterative predict-and-verify approach to locating the *S. cerevisiae* ARSs, using OriDB [37] as a surrogate for the experimental verification process. OriDB has three categories of ARSs: “confirmed”, “likely” and “dubious”. Based on various criteria and independent lines of verification (see Text S1), we classified the 284 “confirmed” and 216 “likely” ARSs as functional ARSs and the “dubious”, as well as any other locations for which we have no data, as nonfunctional ARSs. At the end of 5 iterations of our method, our top 100 distinct predictions (Dataset S5) include 91 verified ARSs, 8 nonfunctional sequences, and 1 “dubious” ARS. According to our definition, this gives a 9% failure rate. In comparison, the top 100 distinct predictions of Oriscan (Dataset S6), which includes 84 functional ARSs, 14 nonfunctional sequences, and 2 “dubious” ones, give a 16% failure rate. Thus our iterative method, which relies only on predicting the ACS, represents more than 44% improvement over Oriscan's top 100 predictions (see Text S1 for details).

We also evaluated the efficacy of our iterative approach by comparing the first 100 predictions from our iterative approach with the top 100 sites predicted from a PWM deduced only from the initial set of 69 *Kl*ARSs (no iterations). The latter, non-iterative, predictions include 63 functional ARSs, 31 candidates that were verified to be non-functional and 6 candidates that could not be or were not verified. Of note is that 4 of the 6 unverified candidates were ranked at the bottom of the list where the ratio of functional to non-functional predictions was 4 to 8. The top 100 predictions from the iterative approach yields 70 functional ARSs, 28 candidates that were verified to be non-functional and 2 candidates that could not be verified. Comparing the ratio of negative to positive prediction we have 0.4 for the iterative method and a higher 0.49 for the non-iterative method. In summary, the iterative approach is superior to other approaches for identifying ARSs of a naive genome both in efficacy, accuracy and scope.

### *KARS12* and its dimeric character

*KARS12* (alias *Kl*ARS503 here) is one of the two genomic *K lactis* ARSs that were known prior to this work and its 50 bp ACS was previously predicted based on the essential element determined by linker-scanning analysis of *KARS101* (alias *Kl*ARS406) [28], [29].

**Figure 6:**
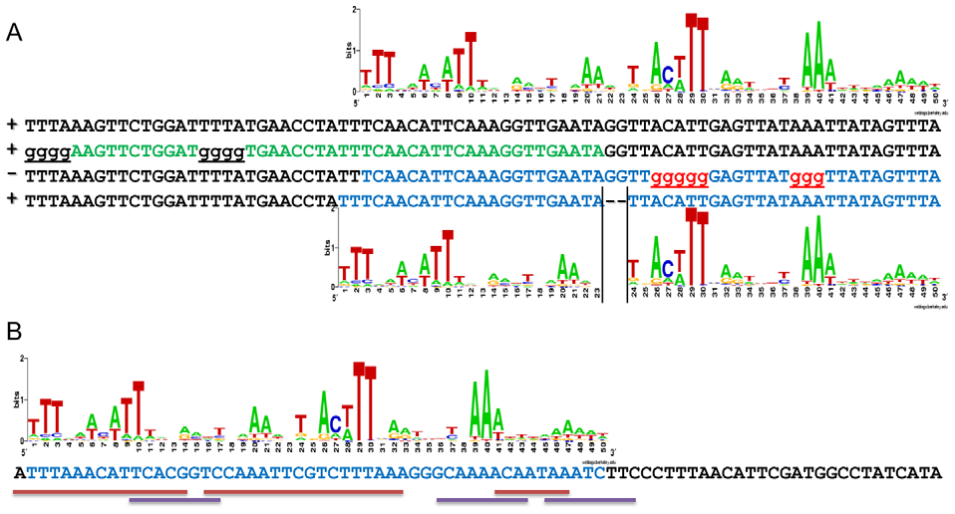
KARS12 and KARS101, two previously identified *Kl*ARSs. (A) Overlapping ACSs of KARS12 (*Kl*ARS503) as predicted previously [29], [30] (colored in green) and in this study (colored in blue). Substitution mutations are shown in lower case font and deletions in dashes. Mutations that disrupt ARS function are highlighted in red, and mutations that do not affect ARS function are highlighted in black. The logo of 50bp ACS is superimposed for reference. (+) indicates ARS function and (−) indicates no ARS function. (B) Complete overlapping ACSs (colored in blue) of KARS101 identified by linker substitution analysis [28] and by motif search in this study. Octanucleotide linker mutations that destroyed ARS function (−) are indicated by red bars and those that compromised ARS function (+/−) are indicated by grey bars. The logo of 50bp ACS is superimposed for reference.

A PWM offers a more refined motif representation than a consensus sequence and it is therefore not surprising that it can better predict putative ACS sites. Specifically, in the case of *KARS12* the previously reported ACS (Figure 6A, highlighted green) scored significantly lower than another overlapping site (Figure 6A, highlighted blue) (4.23 vs. 6.8 SADMAMA scores). Mutational analysis directed to 8 bp that affected only the originally annotated *KARS12* ACS had no effect on the activity of *KARS12* (Figure 6A, black lower case letters) while mutations that replaced 8 bp from the higher scoring predicted ACS destroyed its activity (Figure 6A, red lower case letters).

A closer look at *KARS12* reveals an interesting feature that is unique to this ARS. If we delete the (GG) nucleotides in positions 22–23 we get a significantly better match to the ACS (score 17.6). To see whether this prediction is borne out in an experiment we created another mutant of *KARS12* where we deleted this (GG) dinucleotide (Figure 6A). This mutant was indeed functional, supporting the conjectured dimeric nature of the ACS and suggests further that the *Kl*ORC might have some flexibility in the spacing between the two halves. To further test this hypothesis, we scanned the *K. lactis* genome for other sites whose ACS score is high if we allow a deletion of 1–3 bases at this same position. None of the 11 high scoring such candidates we tested showed ARS activity (see Text S1). To further test whether there is room for flexibility between the two putative parts of the *Kl*ACS we used MAFFT engine in the JALView multiple alignment GUI [51], [52] to realign the extended ACS motif (best match to ACS plus 5 flanking bp on each side). The idea is that if there is a flexible gap between the two parts of the motif the alignment could be improved by inserting the correct gap length. The results were almost identical to aligning the original gapless ACS. In other words, there was no support for a flexible gap in the dimeric structure using the above criteria for flexibility.

The 50-bp ACS of *KARS101* was previously verified by linker-scanning mutagenesis [28] and it coincides with the ACS (*Kl*ARS406) that we predicted computationally, confirming the accuracy of our predictions. However, because of the size of the linkers (8-mer) and their positions, we are unable to decipher the relative importance of the conserved nucleotides within the ACS motif (Figure 6B).

## Discussion

We have developed a novel iterative approach that uses computational predictions to guide experimental verification in mapping the genome-wide locations of replication origins in a naive genome, *K. lactis.* We found that *K. lactis*, similarly to *S. cerevisiae*, uses defined sequences for the initiation of DNA replication. A unique feature of *K. lactis* ARSs is an unusually long ACS of 50bp, which is both necessary and largely sufficient for ARS activity. Compared to the 17 bp ACS of *Sc*ARSs that requires additional auxiliary B elements for function [53], [54], [55], [56], [57], the *Kl*ACS may have incorporated the necessary features of the auxiliary B elements of *S. cerevisiae* in a spatially more restricted fashion. The entire repertoire of non-repetitive intergenic *K. lactis* ARSs is projected to be no more than 156, which is significantly smaller than the 326 verified *S. cerevisiae* ARSs. Considering that these two yeast species have similar genome sizes (12Mb for *S. cerevisiae* and 10.6Mb for *K. lactis*), the reduced number of origins corresponds with an almost two-fold difference in their average replicon size. Given that only a fraction of all origins is used in any cell division cycle in *S. cerevisiae* [58], [59] and *S. pombe* [60], a decreased number of potential replication origins in *K. lactis* should coincide with either an increase in origin efficiency [61], a significant increase in replication fork rate, or a longer S phase. A recent study using DNA combing showed that the actual number of ARSs in a given genome has little bearing on the average replicon size of the replicating genome [62]. Rather, ARSs are potential initiation sites that are activated in a stochastic fashion in individual cells. The number of sites being activated appears to be responsive to the replication stress presented by different growth conditions. A comparison of the replicon sizes of *S. cerevisiae* and *K. lactis* under the same growth condition may provide information about the relative efficiency of origin usage in these two species.

The conjectured dimeric configuration of the *Kl*ACS may also shed some light on the efficiency of usage of the ARSs in these two species. If two, instead of one copy of ORC binds to each *Kl*ACS, it may improve the probability of activation of ARSs in *K. lactis* and a smaller repertoire of ARSs in *K. lactis* would perform just as well as a repertoire twice its size in *S. cerevisiae.* In support, a recent study in *S. pombe* showed that limiting initiation factors regulate the efficiency of replication origin usage [60]. Replication origins that are more accessible or have higher affinity for the limiting initiation factors are preferentially initiated. Conforming to replication origins of other eukaryotes, *K. lactis* ARSs have an extreme bias for intergenic regions but without any bias with respect to the orientation of transcription of the flanking genes. This independence from nearby transcription is consistent with a more robust initiation mechanism [24]. Another possibility for the different densities of replication origins in the two species is that this phenomenon is a remnant of the WGD event. *S. cerevisiae* may have retained the duplicated origins which are also sites of active evolutionary changes, and therefore the lack of synteny of ARSs between these two strains. Future comparative analysis of additional non-WGD strains such as *S. kluyveri* [25] and WGD strains could address this possibility.

Based on the newly derived genome map of *K. lactis* ARSs and the cumulative genome map of *S. cerevisiae* ARSs, we found that there is no evolutionary conservation in the location of nonrepetitive ARSs. Rather, there is a statistically significant preference for ARSs to be located at nonsyntenic intergenic spaces of these two species as previously noted for *S. cerevisiae* [47]. It is unclear whether there are evolutionary forces that exert a positive selection for ARSs to be located at rearranged chromosomal breakpoints, or replication origins are the hotspots for generating chromosomal rearrangements. It is equally intriguing whether some of the syntenically conserved ARSs are positively selected for to serve a specific function as suggested by the invariant position of rDNA initiation sites in eukaryotes. These questions could be addressed by expanding the comparative analysis to include additional genomic replication origin maps of yeast species of various evolutionary distances.

## Materials and Methods

### Construction of *K. lactis* genomic libraries

Genomic DNA from the *uraA* auxotrophic MW98-8C strain of *K. lactis* [31] was isolated using standard methods. Cells were broken using glass bead lysis and the genomic DNA was separated from mitochondrial DNA using a standard CsCl gradient protocol. Following the gradient, DNA was precipitated with 75% ethanol and resuspended in water. Prior to library construction, the DNA was digested with *MboI* and treated with Antarctic Phosphatase (New England Biolabs) to prevent the multimerization of genomic fragments. The digested insert DNA was ligated into the unique *BamHI* site in the pIL07 vector (see below) using T4 DNA Ligase (New England Biolabs). The ligation reaction was purified using a PCR Purification Kit column (QIAgen) and redigested with *BamHI* to linearize all empty pIL07 molecules. The resulting reaction was used to transform chemically competent *E. coli* cells using standard methodology. Colonies from the transformed ligation reaction were pooled and plasmid DNA was extracted using the Wizard Plus SV Miniprep Kit (Promega).

### Construction of pIL07

The pIL07 plasmid was made from the pUC19 subcloning vector for the purpose of isolating ARSs. *S. cerevisiae LEU2* gene was PCR amplified with primers containing *XbaI* sites, digested and ligated into the *XbaI* site in pUC19. *URA3* and *S. cerevisiae CEN5* were cloned similarly into the *EcoRI* and *HindIII* sites respectively. The *BamHI* site used for screening ARS fragments is located between the divergently transcribed *URA3* and *LEU2* genes. Full sequence of this vector is available upon request.

### Screening of *K. lactis* libraries for ARS activity

The libraries were used to transform the MW98-8C strain using a standard Lithium Acetate protocol [31] and plated on medium lacking uracil to select for ARS function. Transformants were individually restreaked onto fresh plates and subsequently grown in culture medium lacking uracil to enrich for the ARS bearing plasmid. The plasmids were isolated from yeast using a modified DNA extraction protocol. 2 mL of culture was pelleted and resuspended in 500 µL of a buffer consisting of 1 M sorbitol, 0.1M EDTA, 1 µL/mL β-mercaptoethanol, and 0.5 mg/mL Yeast Lytic Enzyme (VWR, catalog # IC360951). The suspension was incubated at 37 degrees for 1 hour. The treated cells were pelleted and processed using the Wizard Plus SV Miniprep kit (Promega). 5 µL of the resulting eluate was used to transform *E. coli* and transformants were miniprepped to isolate the ARS-bearing plasmid. A small sample of each plasmid was used to re-transformed MW98-8C to confirm ARS function. Confirmed ARS plasmids were sequenced using primers IL325 (5′-GCCAAACAACCAATTACTTGTTGAGA-3′) and IL326 (5′-TTCGTTGCTTGTCTTCCCTAGTTTC-3′) from both ends of the ARS fragment.

### Cloning, truncation, and mutagenesis of ARS sequences

To clone and test predicted regions of DNA for ARS activity, primers containing *BamHI* and *BglII* cloning sites were designed to anneal to the relevant region. PCR amplified DNA was cloned into pIL07 and confirmed by sequencing. Several clones of each predicted region were used to transform MW98-8C to test for ARS function. ARS fragments were truncated by amplifying and cloning smaller fragments of the ARS region. Site-directed mutagenesis was performed using a fusion-PCR mutagenesis method [63]. The DNA region to be mutagenized was PCR amplified in two separate fragments, which overlap by 50–60 base pairs. The overlap primers contained the desired mutation. After the initial PCR, the two fragments were purified separately and used together in another PCR reaction without any template DNA. The overlapping regions in the two DNA fragments acted as primers for each other and PCR produced a final molecule which contained the entire DNA fragment including the mutation of interest. This fragment was then cloned into the vector and sequenced as above.

### Two-dimensional DNA gel electrophoresis

Two-dimensional DNA gel electrophoresis was performed according to the neutral–neutral method [64]. Purified DNA was digested to completion with *Nco*I. To enrich the sample for replicating DNA, digested DNA was passed through BND cellulose (Sigma-Aldrich) columns as described in [65]. ARS probes were made by amplifying 1.5 kb region centered at the ARS by PCR and radiolabeled with [α-^32^P] dATP using the Prime-It II Random primer labeling kit from Stratagene.

### Iterative predict-and-verify approach to identifying *K. lactis* ARSs

The gibbsMarkov part of GIMSAN was applied to the set of previously verified ARSs using a training file consisting of all *K. lactis* intergenic regions with the parameters: (- gibbsamp -l 50 -p 0.05 -best_ent -em 0 -t 200 -L 200 -ds -markov 4). In addition to the PWM reported by GIMSAN, SADMAMA was given the same training file as GIMSAN, which was also used as the input target set for scanning for matches to the PWM. Matches at repetitive telomeric regions were ignored. Other parameters were: (-printScoresGTT first_set -siteThresholdLearnedFrom 1e-4 both_strands nullTrainFile -tests – -pwmPC 0.01 -m 4 both_strands -siteNullScore avg_strands). The *K. lactis* genome downloaded from NCBI is (ACCESSION NC_006037 VERSION NC_006037.1 GI:50313006). Intergenic sequences were generated from the GenBank files of the genome by filtering out all sequences with feature type ‘gene’, ‘LTR’, or ‘gap’.

The fifth iterative list of *K. lactis* candidates was compiled from the set of ARSs that were verified in the previous 4 steps with one exception. A telomeric ARS (C - 44929) was removed, as its similarity to the originally screened telomeric ARS at the end of chromosome C would have biased the estimated PWM. As it was designed to be the last iteration this 5th list contained 41 candidates.

### Other techniques for predicting ARSs

To complement our main iterative approach to predicting *K. lactis* ARSs we tried several other prediction techniques. Excluding the overlap with the iterative procedure the net result of these predictions was 4 verified ARSs and 32 candidates that were verified to be non-functional. Briefly, the following techniques were used:

- Predicting ARSs based on the PWMs derived from our LOO procedure to estimate the number of ARSs. These were more geared toward improving that estimate than to actually finding new ARSs.
- Looking for high scoring candidates in *coding* regions (all other methods including the main iterative one scanned only intergenic regions.
- Favoring predictions (i.e., willing to tolerate lower scores) that fall in long stretches that are void of ARSs.
- Using a specially modified gapped version of SADMAMA we looked for dimeric matches that have a flexible number of spacers between the two main parts of the ACS PWM.
- Using the same gapped SADMAMA we tried scoring matches by ignoring weak columns in the 50 bp ACS PWM.

### ARS conservation analysis

Our conservation analysis hinges on the analysis of syntenic pairs of *S. cerevisiae* and *K. lactis* protein coding genes. The latter are defined as follows: a pair of *lactis-cerevisiae* genes is declared “homologous” if there exists a pairwise alignment of the two with BLAST E-value <1e-4. Two pairs of adjacent *K. lactis* and *S. cerevisiae* genes are declared syntenically conserved if the two pairs of genes are pairwise homologous and if their orientations are syntenically conserved. Note that a pair of *K. lactis* genes can thus be syntenically conserved with more than one pair of *S. cerevisiae* genes and vice versa. Fortunately, these cases do not occur often: the 2018 syntenic pairs are made of 1992 distinct *S. cerevisiae* pairs of genes and 1988 distinct *K. lactis* pairs. By extension, we consider *S. cerevisiae* and *K. lactis* intergenic regions are syntenically conserved if their corresponding flanking genes are.

Our list of intergenic *Kl*ARSs consists of 145 sequences identified as described above (Table S1). There is however some freedom in defining the set of *cerevisiae* intergenic ARSs. In particular, OriDB lists “Confirmed”, “Likely”, and “Dubious” ARSs that are subject to judicious classification. To minimize the potential effects of this “judicious classification” on our analysis we performed the analysis twice: first with essentially only the confirmed OriDB ARSs and then again when we added high scoring likely OriDB ARSs. Reassuringly, the results were essentially the same (see more details in Text S1).

### Flanking gene strand statistics

To determine the strand statistics of the pairs of genes flanking the ARSs in both species we used the following criteria. For *K. lactis* we positioned the 145 intergenic ARSs based on the center position of their best ACS match. The strand of the flanking pair of genes was determined from the annotations in the *K. lactis* GenBank files mentioned above. For *S. cerevisiae* we made use of a list of 298 verified intergenic ARSs for which we identified an intergenic best ACS match (all ARSs with an ARS type different than ‘Likely’ in Dataset S7, see section on *ARS positional conservation analysis* in Text S1). List of adjacent genes and their strands was compiled from SGD's latest version of the *S. cerevisiae* protein coding sequences as of Jun 6th 2008. These genes were then mapped back to the October 2003 genome (NC_001133.3 GI:37362608 - NC_001148.2 GI:37362698) based on shared systematic name.

## Supporting Information

Dataset S1 List of 123 predicted KlACS compiled from 5 systematic iterations of predict-and-verify (the 5th cycle consisted of 41 predictions). The 'functional' column refers to the ARS functionality of the region defined by the 50bp predicted ACS plus the flanking 200bp. We could not clone five of the predicted ARSs (no entry under the 'functional' column). The first of these in particular (3 - 16994) resisted multiple cloning attempts. Interestingly, it is very close to start of chromosome C and it joins other lines of evidence indicating some mismatch in the telomeric regions between our strain's genome and the published one. Of the 118 predicted ARSs that we could clone, 75 exhibited ARS activity while 43 did not. Another 4 IGLARS were predicted and verified using other, less productive predictions (see Dataset S2).
(0.01 MB CSV)

Dataset S2 List of additional 36 predicted KlACS using a multiple of other techniques to computationally predict ARSs such as, favoring predictions that fall in long ARS-less stretches, scoring matches by ignoring weak columns, using cross validation techniques and looking for "dimeric matches" (matches that have a flexible number of spacers). The yield here was much lower: 4 verified functional versus 32 verified as non-functional.
(0.01 MB CSV)

Dataset S3 The 50bp-wide PWM derived from the list of 148 verified ARSs with the exception of two telomeric ARSs (E/5 - 2231306 and F/6 - 2599876) whose similarity to the telomeric ARS at the end of chromosome C would have biased the estimated PWM. Similarly, the KARS12 was left out due to the suspected dimeric character of its ACS match.
(0.01 MB TXT)

Dataset S4 Summary of some of the ARS positional conservation analysis.
(0.04 MB DOC)

Dataset S5 List of 100 top ARS predictions compiled from 5 iterations of predict-and-verify starting with Breier et al.'s training set of 26 sequences and PWM (see details above). The last 5 columns are taken from OriDB based on coordinate matching. The 4 exceptions to the last statement are when under 'ars_type' are either 'no' (meaning this ARS was verified as non-functional) or 'predicted-yes' (meaning this ARS was verified to be functional). Note that all 4 'predicted-yes' ARS are known likely OriDB ARSs only our versions here are significantly shorter (see also Dataset S8).
(0.01 MB CSV)

Dataset S6 List of top 100 distinct ARS predictions compiled from Breier et al.'s original list of top 125 candidates. In addition to removing ARSs belonging to the excluded regions (see Text S1) we also noted that 6 candidate ARSs on the original appear twice: ARS1424 (42,104), ARS1109 (4,23), ARS1621 (20,91), ARS1628 (66,73), ARS702 (25,100) and (81,105) that are 24bp apart. There are seemingly 15 candidate ARSs whose functionality is unknown, however a closer look reveals that (117) Ch. 3, 271748 is probably a shifted coordinate version of ARS316 and should be counted as a correct prediction. Again, (102) is in fact a likely OriDB ARS. This leaves us with 14 predictions whose functionality is unknown and 2 dubious for a total of 16 failures.
(0.01 MB CSV)

Dataset S7 List of 344 verified and high scoring likely *S. cerevisiae* intergenic ARSs. The ACS coordinates, strand and score refer to the predicted best match to a 35bp long ACS PWM as explained in the text. An ARS is classified as intergenic based on the center position of this match. ARSs with type "predicted" belong the list of ARSs we predicted based on their high scoring ACS match (Dataset S8) whereas type "seed_Ivan" refers to ARSs we randomly isolated in a screen (Dataset S9).
(0.02 MB CSV)

Dataset S8 Verification of 16 top high scoring ACS matches in the *S. cerevisiae* intergenic regions that excluded all confirmed ARSs. The last 4 columns are taken from OriDB based on matching coordinates. The 'functional' column refers to the ARS functionality of the 33bp predicted ACS taken with the flanking 200bp on each side. Of note: all 7 of predicted ARSs that coincide with likely OriDB ARS are functional, 2 more (Ch. 2 417582 & 591418) are most likely corrections to the respective coordinates of ARS215 and ARS219, 2 others (Ch.5 192693 & Ch.8 94959) are brand new ARSs we report here, while the remaining 5 are not functional. All ACS scores were based on a preliminary, width 33, PWM that was generated by GIMSAN from the list of confirmed OriDB ARSs sans all but one core X ARSs. This PWM was then further manually modified to remove all "non-interesting" columns and was used to scan the *S. cerevisiae* intergenic regions using a modified version of SADMAMA that allows gapped positions in the motif.
(0.01 MB CSV)

Dataset S9 Table of 34 distinct ARSs isolated in a random screen of *S. cerevisiae* ARSs. Columns 2-4 are derived from blasting the ARS sequence against the *S. cerevisiae* genome (October 2003). The last 4 columns are taken from OriDB based on matching coordinates. Of note: 8 of the screened ARSs coincide with likely OriDB ARSs, 24 coincide with confirmed OriDB ARSs, one is chimeric (303-100-1) and another (303-7-1), a novel ARS we report here.
(0.01 MB CSV)

Dataset S10 List of 54 "excluded" regions that were avoided in the prediction process. These consist of two large families of nearly identical ARSs: subtelomeric X-ARS and an ARS family which is telomeric to it (see text above), as well as a few other pairs of nearly identical ARSs of which one ARS from each pair was excluded (see 'reason' column). The last 5 columns are taken from OriDB based on matching coordinates.
(0.01 MB CSV)

Dataset S11 Newly identified and better localized *S. cerevisiae* ARSs. The first ARS was identified through a random screen while the other nine through high scoring ACS matches followed by verification of the flanking segments. Of these nine, two are completely new while seven other offer verification and localized versions of more loosely defined likely ARSs. Note that 6 additional likely OriDB ARSs were verified in our random screen but they are not as compact as the predicted ones included here (Dataset S9).
(0.01 MB TXT)

Table S1 List of all *K. lactis* ARSs.
(0.01 MB CSV)

Text S1 Supplementary computational methods.
(0.13 MB DOC)

## References

[1] Bell S, Stillman B (1992). ATP-dependent recognition of eukaryotic origins of DNA replication by a multiprotein complex.. Nature.

[2] Hamlin JL, Mesner LD, Lar O, Torres R, Chodaparambil SV (2008). A revisionist replicon model for higher eukaryotic genomes.. J Cell Biochem.

[3] Sclafani RA, Holzen TM (2007). Cell cycle regulation of DNA replication.. Annu Rev Genet.

[4] Struhl K, Stinchcomb DT, Scherer S, Davis RW (1979). High-frequency transformation of yeast: autonomous replication of hybrid DNA molecules.. Proc Natl Acad Sci U S A.

[5] Chan CS, Tye BK (1980). Autonomously replicating sequences in *Saccharomyces cerevisiae*.. Proc Natl Acad Sci U S A.

[6] van Houten JV, Newlon CS (1990). Mutational analysis of the consensus sequence of a replication origin from yeast chromosome III.. Mol Cell Biol.

[7] Diffley JFX, Cocker JH (1992). Protein-DNA interactions at a yeast replication origin.. Nature.

[8] Segurado M, de Luis A, Antequera F (2003). Genome-wide distribution of DNA replication origins at A+T-rich islands in *Schizosaccharomyces pombe*.. EMBO Rep.

[9] Dai J, Chuang RY, Kelly TJ (2005). DNA replication origins in the *Schizosaccharomyces pombe* genome.. Proc Natl Acad Sci U S A.

[10] Houchens CR, Lu W, Chuang RY, Frattini MG, Fuller A (2008). Multiple mechanisms contribute to *Schizosaccharomyces pombe* origin recognition complex-DNA interactions.. J Biol Chem.

[11] Patel PK, Arcangioli B, Baker SP, Bensimon A, Rhind N (2006). DNA replication origins fire stochastically in fission yeast.. Mol Biol Cell.

[12] Heichinger C, Penkett CJ, Bahler J, Nurse P (2006). Genome-wide characterization of fission yeast DNA replication origins.. Embo J.

[13] Hayashi M, Katou Y, Itoh T, Tazumi A, Yamada Y (2007). Genome-wide localization of pre-RC sites and identification of replication origins in fission yeast.. Embo J.

[14] Feng W, Collingwood D, Boeck ME, Fox LA, Alvino GM (2006). Genomic mapping of single-stranded DNA in hydroxyurea-challenged yeasts identifies origins of replication.. Nat Cell Biol.

[15] Blumenthal A, Kriegstein HJ, Hogness DS (1973). The units of DNA replication in *Drosophila melanogaster* chromosomes.. Cold Spring Harbor Symp Quant Biol.

[16] Walter J, Newport JW (1997). Regulation of replicon size in *Xenopus* egg extracts.. Science.

[17] Aladjem MI, Fanning E (2004). The replicon revisited: an old model learns new tricks in metazoan chromosomes.. EMBO Rep.

[18] Shirahige K, Iwasaki T, Rashid M, Ogasawara N, Yoshikawa H (1993). Location and characterization of autonomously replicating sequences from chromosome VI of *Saccharomyces cerevisiae*.. Mol Cell Biol.

[19] Wyrick JJ, Aparicio JG, Chen T, Barnett JD, Jennings EG (2001). Genome-wide distribution of ORC and MCM proteins in *S. cerevisiae*: high-resolution mapping of replication origins.. Science.

[20] Raghuraman MK, Winzeler EA, Collingwood D, Hunt S, Wodicka L (2001). Replication dynamics of the yeast genome.. Science.

[21] Yabuki N, Terashima H, Kitada K (2002). Mapping of early firing origins on a replication profile of budding yeast.. Gene Cells.

[22] Breier AM, Chatterji S, Cozzarelli NR (2004). Prediction of *Saccharomyces cerevisiae* replication origins.. Genome Biol.

[23] Nieduszynski CA, Knox Y, Donaldson AD (2006). Genome-wide identification of replication origins in yeast by comparative genomics.. Genes Dev.

[24] Donato JJ, Chung SC, Tye BK (2006). Genome-wide hierarchy of replication origin usage in *Saccharomyces cerevisiae*.. PLoS Genet.

[25] Payen C, Fischer G, Marck C, Proux C, Sherman DJ (2009). Unusual composition of a yeast chromosome arm is associated with its delayed replication.. Genome Res.

[26] Wolfe KH, Shields DC (1997). Molecular evidence for an ancient duplication of the entire yeast genome.. Nature.

[27] Fitzpatrick DA, Logue ME, Stajich JE, Butler G (2006). A fungal phylogeny based on 42 complete genomes derived from supertree and combined gene analysis.. BMC Evol Biol.

[28] Fabiani L, Frontali L, Newlon CS (1996). Identification of an essential core element and stimulatory sequences in a *Kluyveromyces lactis* ARS element, KARS101.. Mol Microbiol.

[29] Irene C, Maciariello C, Micheli G, Theis JF, Newlon CS (2007). DNA elements modulating the KARS12 chromosomal replicator in *Kluyveromyces lactis*.. Mol Genet Genomics.

[30] Irene C, Maciariello C, Cioci F, Camilloni G, Newlon CS (2004). Identification of the sequences required for chromosomal replicator function in *Kluyveromyces lactis*.. Mol Microbiol.

[31] Bianchi MM, Falcone C, Chen XR, Weslowski-Louvel M, Frontali L (1987). Transformation of the yeast *Kluyveromyces lactis* by new vectors derived from the 1.6 µm circular plasmid pKD1.. Curr Genet.

[32] Schaffer AA, Aravind L, Madden TL, Shavirin S, Spouge JL (2001). Improving the accuracy of PSI-BLAST protein database searches with composition-based statistics and other refinements.. Nucleic Acids Res.

[33] Ng P, Keich U (2008). GIMSAN: a Gibbs motif finder with significance analysis.. Bioinformatics.

[34] Keich U, Gao H, Garretson JS, Bhaskar A, Liachko I (2008). Computational detection of significant variation in binding affinity across two sets of sequences with application to the analysis of replication origins in yeast.. BMC Bioinformatics.

[35] Kohavi R (1995). A study of cross-validation and bootstrap for accuracy estimation and model selection.. Proceedings of the Fourteenth International Joint Conference on Artificial Intelligence.

[36] Bhaskar A, Keich U (2010). Confidently estimating the number of DNA replication origins.. submitted.

[37] Nieduszynski CA, Hiraga S, Ak P, Benham CJ, Donaldson AD (2007). OriDB: a DNA replication origin database.. Nucleic Acids Res.

[38] Brewer BJ, Fangman WL (1991). Mapping replication origins in yeast chromosomes.. BioEssays.

[39] Shore D, Stillman DJ, Brand AH, Nasmyth KA (1987). Identification of silencer binding proteins from yeast: possible roles in SIR control and DNA replication.. Embo J.

[40] McNally FJ, Rine J (1991). A synthetic silencer mediates SIR-dependent functions in *Saccharomyces cerevisiae*.. Mol Cell Biol.

[41] Grewal SI, Klar AJ (1997). A recombinationally repressed region between mat2 and mat3 loci shares homology to centromeric repeats and regulates directionality of mating-type switching in fission yeast.. Genetics.

[42] Nakayama J, Allshire RC, Klar AJ, Grewal SI (2001). A role for DNA polymerase alpha in epigenetic control of transcriptional silencing in fission yeast.. Embo J.

[43] Linskens MH, Huberman JA (1988). Organization of replication of ribosomal DNA in *Saccharomyces cerevisiae*.. Mol Cell Biol.

[44] Sanchez JA, Kim SM, Huberman JA (1998). Ribosomal DNA replication in the fission yeast, *Schizosaccharomyces pombe*.. Exp Cell Res.

[45] Gogel E, Langst G, Grummt I, Kunkel E, Grummt F (1996). Mapping of replication initiation sites in the mouse ribosomal gene cluster.. Chromosoma.

[46] Yoon Y, Sanchez JA, Brun C, Huberman JA (1995). Mapping of replication initiation sites in human ribosomal DNA by nascent-strand abundance analysis.. Mol Cell Biol.

[47] Gordon JL, Byrne KP, Wolfe KH (2009). Additions, losses, and rearrangements on the evolutionary route from a reconstructed ancestor to the modern *Saccharomyces cerevisiae* genome.. PLoS Genet.

[48] Di Rienzi SC, Collingwood D, Raghuraman MK, Brewer BJ (2009). Fragile genomic sites are associated with origins of replication.. Genome Biol Evol 2009.

[49] Dietrich FS, Voegeli S, Brachat S, Lerch A, Gates K (2004). The *Ashbya gossypii* genome as a tool for mapping the ancient *Saccharomyces cerevisiae* genome.. Science.

[50] Kellis M, Birren BW, Lander ES (2004). Proof and evolutionary analysis of ancient genome duplication in the yeast *Saccharomyces cerevisiae*.. Nature.

[51] Katoh K, Misawa K, Kuma K, Miyata T (2002). MAFFT: a novel method for rapid multiple sequence alignment based on fast Fourier transform.. Nucleic Acids Res.

[52] Clamp M, Cuff J, Searle SM, Barton GJ (2004). The Jalview Java alignment editor.. Bioinformatics.

[53] Srienc F, Bailey JE, Campbell JL (1985). Effect of ARS1 mutations on chromosome stability in *Saccharomyces cerevisiae*.. Mol Cell Biol.

[54] Holmes SG, Smith MM (1989). Interaction of the H4 autonomously replicating sequence core consensus sequence and its 3′-flanking domain.. Mol Cell Biol.

[55] Walker SS, Francesconi SC, Eisenberg S (1990). A DNA replication enhancer in *Saccharomyces cerevisiae*.. Proc NatlAcad Sci.

[56] Celniker SE, Sweder K, Srienc F, Bailey JE, Campbell JL (1984). Deletion mutations affecting autonomously replicating sequence ARS1 of *Saccharomyces cerevisiae*.. Mol Cell Biol.

[57] Deshpande AM, Newlon CS (1992). The ARS consensus sequence is required for chromosomal origin function in *Saccharomyces cerevisiae*.. Mol Cell Biol.

[58] Friedman KL, Brewer BJ, Fangman WL (1997). Replication profile of *Saccharomyces cerevisiae* chromosome VI.. Genes Cells.

[59] Yamashita M, Hori Y, Shinomiya T, Obuse C, Tsurimoto T (1997). The efficiency and timing of initiation of replication of multiple replicons of *Saccharomyces cerevisiae* chromosome VI.. Genes Cells.

[60] Wu PY, Nurse P (2009). Establishing the program of origin firing during S phase in fission Yeast.. Cell.

[61] Poloumienko A, Dershowitz A, De J, Newlon CS (2001). Completion of replication map of *Saccharomyces cerevisiae* chromosome III.. Mol Biol Cell.

[62] Tuduri S, Tourriere H, Pasero P (2010). Defining replication origin efficiency using DNA fiber assays.. Chromosome Res.

[63] Erdeniz N, Mortensen UH, Rothstein R (1997). Cloning-free PCR-based allele replacement methods.. Genome Res.

[64] Brewer BJ, Fangman WL (1987). The localization of replication origins on *ARS* plasmids in *S. cerevisiae*.. Cell.

[65] Dijkwel PA, Vaughn JP, Hamlin JL (1991). Mapping of replication initiation sites in mammalian genomes by two-dimensional gel analysis: stabilization and enrichment of replication intermediates by isolation on the nuclear matrix.. Mol Cell Biol.

[66] Crooks GE, Hon G, Chandonia JM, Brenner SE (2004). WebLogo: a sequence logo generator.. Genome Res.

