# Supplementary material for: A Comprehensive Genome-Wide Map of Autonomously Replicating Sequences in a Naive Genome
Source: PLoS Genet. 2010 May 13;6(5):e1000946. doi: 10.1371/journal.pgen.1000946 (PMC2869322; doi:10.1371/journal.pgen.1000946)
Supplement: Dataset S4 — Summary of some of the ARS positional conservation analysis. (0.04 MB DOC) [file pgen.1000946.s004.doc]

| Feature | No. of balls | No. of red balls | No. of balls in sample | No. of red balls in sample | two-sided P-value (verdict) |
| --- | --- | --- | --- | --- | --- |
| *S. cerevisiae* IG ARSs in syntenic regions[[1]](#footnote-2) | 3245828 *S. cerevisiae* intergenic bps[[2]](#footnote-3) | 793058 syntenic intergenic bps | 298 verified intergenic cerevisiae ARSs | 51 of these ARSs are in syntenic regions | 0.003  (Number of red balls in the *sample* is) under-represented: 0.24 vs. 0.17 |
| IG verified and high-scoring “likely” ARSs in syntenic regions | same | same | 344 verified and high scoring “likely” ARSs | 56 of these are flanked by syntenic pairs | 0.0003  under-represented: 0.24 vs. 0.16 |
| *K. lactis* IG ARSs in syntenic regions | 3173528 *K. lactis* IG bps | 1077128 bps in *K. lactis* syntenic IG regions | 145 IG *K. lactis* ARSs | 37 of these lie in syntenic regions | 0.04  borderline under-represented: 0.34 vs. 0.26 |
| *S. cerevisiae* ARSs in IG regions syntenic to ARS-containing *K. lactis* IG regions | 1992 *S. cerevisiae* syntenic intergenic regions | 51 of these regions that contain an *S. cerevisiae* ARS | 38 *S. cerevisiae* IG regions that are syntenic to *K. lactis* IG regions containing an ARS | 2 of these *S. cerevisiae* regions contain an ARS | 0.51  essentially random:  0.03 vs. 0.05 |
| *S. cerevisiae* verified and high-scoring likely ARSs in IG regions syntenic to ARS-containing *K. lactis* IG regions | same | 56 of these regions that contain a verified or high-scoring likely *S. cerevisiae* ARS | same | 2 of these *S. cerevisiae* regions contain a verified or high-scoring likely ARS | 0.58  essentially random:  0.028 vs. 0.053 |

1. Syntenic intergenic regions are the intergenic regions flanked by syntenic pairs of genes. [↑](#footnote-ref-2)
2. Except for the ones that overlap the ARSs that were filtered for duplications. [↑](#footnote-ref-3)
