# Supplementary material for: A Comprehensive Genome-Wide Map of Autonomously Replicating Sequences in a Naive Genome
Source: PLoS Genet. 2010 May 13;6(5):e1000946. doi: 10.1371/journal.pgen.1000946 (PMC2869322; doi:10.1371/journal.pgen.1000946)
Supplement: Text S1 — Supplementary computational methods. (0.13 MB DOC) [file pgen.1000946.s013.doc]

**Text S1 - Supplementary Computational Methods**

**ARS positional conservation analysis**

We ask the following three questions:

- Is there a significant enrichment/depletion of *cerevisiae* ARSs among the intergenic *cerevisiae* regions that are syntenic to ARS bearing *lactis* intergenic regions?
- Is there a significant enrichment/depletion of *cerevisiae* ARSs among all syntenic *cerevisiae* intergenic regions?
- Is there a significant enrichment/depletion of *lactis* ARSs among all syntenic *lactis* intergenic regions?

We answered all three questions using two-sided hypergeometric tests as detailed below. In particular we find no essential difference between performing the analysis with the more inclusive or more exclusive list of *cerevisiae* intergenic ARSs described below.

**Technical Details**

We downloaded from SGD the latest version of the *cerevisiae* protein coding sequences as of June 6th 2008 (orf_trans.20080606.fasta.gz) and created a BLAST database out of it. We then extracted coding sequences from the *lactis* GenBank files with accession and version headers ACCESSION NC_006037 VERSION NC_006037.1 GI:50313006 and blasted them using “blastp –n -E 1e-4” against the aforementioned *cerevisiae* coding sequences. This gave us the list of “homologous” *cerevisiae*-*lactis* pairs that was then processed further to find mutually adjacent pairs of such homologous pairs that conserve the relative orientation (“syntenic pairs”). Finally the *cerevisiae* genes were mapped back to the October 2003 version of the genome (NC_001133.3 GI:37362608 - NC_001148.2 GI:37362698) based on their systematic names.

The more exclusive, verified list of *cerevisiae* ARSs was compiled as follows. Starting with the 325 confirmed OriDB ARSs we removed from the list all the 14 Core X ARSs except for one, ARS400. This was done since these ARSs are part of a fairly long subtelomeric repeat that could bias our analysis. For the same reason ARS601, which has a significant overlap with ARS602, was removed, as was the telomeric ARS1001 that is “too similar” to ARS200. ARS403 exactly matches a contiguous block of ARS1023 we therefore dropped the latter. This left us with 309 confirmed OriDB ARSs, two of which, ARS215 and ARS219, we replaced with our own verified versions (see **Dataset S**8). We then added 15 additional ARSs that we verified as functional (for a total of 324 verified ARSs), of which 12 intersect likely OriDB ARSs and the other 3 are brand new (see also the discussion below on *cerevisiae* ARSs as well as **Dataset** S9).

By *cerevisiae* intergenic verified ARSs we mean the subset of 298 of the abovementioned 324 verified ARSs for which the central position of the best match to the ACS PWM is located between two adjacent *cerevisiae* genes. This ACS PWM was generated by first applying the gibbsMarkov part of GIMSAN to the 324 verified ARSs (‘-l 35 -gibbsamp -best_clr -t 200 -L 200 -ds -markov 4 -p 0.05 -em 0’). Two columns of low information content were masked and the “gapped” PWM was used by a modified version of SADMAMA (allowing gapped positions in the PWM) to determine the best match in each ARS (‘-printScoresGTT first_set -siteThresholdLearnedFrom 1e-3 both_strands nullTrainFile -tests -- -pwmPC 0.01 -m 4 both_strands -siteNullScore avg_strands’). A rather lenient threshold of ‘–t 1e-3’ was given to SADMAMA so as to avoid making an arbitrary selection in the few ARS sequences that seem to lack any good ACS match. We used the same *cerevisiae* intergenic background file for both GIMSAN and SADMAMA generated from the GenBank files of the October 2003 genome by filtering out all sequences with feature type 'gene', 'LTR', or 'gap'.

The more inclusive set of *cerevisiae* ARSs was generated by adding high scoring likely OriDB ARSs to the set of verified ARSs: stating with 95 (out of 242) likely OriDB ARSs that included an ACS score >= 8 (determined by SADMAMA exactly as above) we removed 22 repetitive telomeric and subtelomeric ARSs (**Dataset** S10). Further removing from this list all ARSs that intersect our list of 15 newly verified ARSs left us with 63 high scoring likely OriDB ARSs. Using the same definitions above we find that 46 of these ARSs are intergenic thus, combined with the set of verified ARSs this yields 344 intergenic ARSs (See **Dataset** S7).

We determine the significance of the enrichment/depletion of *cerevisiae* ARSs among all syntenic *cerevisiae* intergenic regions as follows. Under the first model we have 5736 "balls": intergenic *cerevisiae* regions that do no intersect our list of excluded/repetitive *cerevisiae* ARSs. Of these, 1992 are "syntenic balls" which represent that many *cerevisiae* intergenic regions that are flanked by a syntenically conserved pair of genes. Our sample has 294 balls representing that many intergenic regions that are occupied by the 298 distinct verified intergenic ARSs. Of these 294 balls, 51 turn out to be syntenic balls. Since a-priori we do not know whether to expect a relatively small or large number of syntenic balls we should use a two sided test or p-value (2.0e-11). When the more inclusive list of ARSs is used there are 340 balls, or ARS-including intergenic regions, in the sample out of which 56 are syntenic balls (two sided p-value of 2.0e-14).

There is however a possible flaw in this first null model that becomes obvious when one realizes that the average length of the intergenic regions that are syntenically conserved is 398bp while the average length of the intergenic regions that are not syntenically conserved is 655bp. To account for this variation, our second model assumes that the 298 verified intergenic *cerevisiae* ARSs are randomly positioned uniformly across the genome by picking intergenic positions that would serve as the centers of their ACS. Using this model we can again use the hypergeometric distribution only now the 298 balls (verified intergenic *cerevisiae* ARSs) are sampled from an urn containing 3245828 "intergenic balls" (all *cerevisiae* intergenic positions except for the ones which overlap the ARSs that were filtered for duplications) of which 793058 are "syntenic balls" (intergenic positions that are flanked by syntenically conserved genes). We find that 51 of our 298 balls are syntenic yielding a more modest, but still significant, two-sided p-value of 0.003. Using the more inclusive list of ARSs our sample is of 344 balls and 56 of those are syntenic yielding a two-sided p-value of 0.0003 (see also **Dataset** S4).

We next apply these two models to assess the analogous question of the significance of enrichment/depletion of *lactis* ARSs among all *lactis* intergenic regions that are syntenically conserved. Using the first model we find that our sample of 145 balls (ARS bearing intergenic *lactis* regions) from an urn with 5165 "intergenic balls", 1988 of which are "syntenic balls", pulls out 37 syntenic balls (two-sided p-value of 0.001). For the second model we have 3173528 intergenic balls (positions) of which 1077128 are syntenic. Again, our sample of 145 ARSs yields 37 syntenic balls corresponding to a borderline but still significant two-sided p-value of 0.037.

Finally, we would like to determine the significance of the enrichment/depletion of *cerevisiae* ARSs among the intergenic *cerevisiae* regions that are syntenic to ARS-bearing *lactis* intergenic regions. Thus, we concentrate on the 37 intergenic *lactis* ARSs that are syntenically paired with 38 distinct *cerevisiae* pairs of genes (one of the *lactis* pairs syntenically matches two distinct *cerevisiae* pairs). Once again we look at it as a hypergeometric test where in this case we have 1992 balls (number of distinct *cerevisiae* pairs of genes that are syntenically conserved) out of which 51 are red (those pairs of genes that flank a *cerevisiae* ARS). We draw a sample of 38 balls (the number of *cerevisiae* pairs that are syntenic to the genes flanking the aforementioned 37 *lactis* ARSs) and find that 2 of those are red (i.e., the *lactis* and *cerevisiae* ARSs are conserved). The two-sided p-value is an insignificant 0.51. Repeating the analysis with the more inclusive list of ARSs does not change the picture: there are still only 2 ARSs that are syntenically conserved but now this is contrasted with 56 such high-scoring likely or verified *cerevisiae* ARSs that fall within the 1992 syntenic intergenic regions of *cerevisiae* (two sided hypergeometric p-value of 0.58).

**On previous co-localization analyses of *S. cerevisiae* ARSs**

Gordon et al. first reported on the association between *S. cerevisiae* replication origins and chromosomal breakpoints [1]. Our analysis above was independent of theirs, however, we note that their significance analysis is essentially based on the random intergenic region null model that is similar to our first, possibly flawed, model mentioned above. In particular, it does not take into account that the average length of *S. cerevisiae* intergenic regions that include an ARS is considerably bigger than the average length of intergenic regions that do not include any ARS as detailed next.

As explained above, in these co-localization studies it is important to remove from consideration ARSs that are part of long repeats. After removing those duplicates, exactly as described in our ARS conservation analysis above, we ended up with 280 confirmed OriDB intergenic ARSs, where an ARS was considered intergenic if the mid-point of its highest scoring predicted ACS fell in an intergenic region. Using the same list of *cerevisiae* neighboring protein coding genes mentioned above we determined that the average length of the intergenic region flanking a highest scoring ACS match is 1232bp. The remaining 5460 intergenic regions, which do not include a highest scoring ACS match in a confirmed ARS, have a much smaller average length of 532bp.

While our analysis above seems to confirm the conclusion of Gordon et al. in this case we stress that the use of the “intergenic regions” null model (as opposed to intergenic positions) might lead to erroneous conclusions. Their analysis of the co-localization of ARSs and tRNAs might be an example of such a case. Indeed, Gordon et al., and before them Wyrick et al. [2], report finding statistically very significant association between *cerevisiae* ARSs and tRNAs. In both cases this was done on the intergenic regions null model according to which, a feature, say a tRNA or an ARS, is randomly thrown at the intergenic regions. Again, this ignores biases in lengths: not only, as explained above, are ARS-bearing intergenic regions longer than ARS-barren intergenic regions but also tRNA-bearing intergenic regions are even longer on average than the intergenic regions which are void of tRNAs as detailed next.

Relying on the annotations of the October 2003 genbank files alluded to above we identified 252 pairs of adjacent protein coding genes whose intergenic space include the mid point of a tRNA gene. The average length of these intergenic regions is 1709bp. This is in sharp contrast to the average length of 495bp of the remaining 5562 intergenic regions.

**Co-localization of ARSs with tRNAs in *S. cerevisiae* and *K. lactis***

To assess the significance of the co-localization between ARSs and tRNAs we again used the hypergeometric test. Starting with *S. cerevisiae* and using the first, intergenic regions, null model we have a total of 5814 intergenic balls of which 252 are “tRNA balls” (i.e., they contain a tRNA). Our sample of 280 “ARS balls” picked up 38 of these 252 tRNA balls yielding a very significant 2-sided p-value of 3e-10. Note that this is consistent with the previously reported significant p-values of 1.7e-13 [2] and 4.4e-6 [1]. However, when we switch to the intergenic position null model we have 3245828 intergenic balls of which 430580 are tRNA balls (number of positions that are part of a tRNA bearing intergenic region). Our sample of 284 ARS balls[[1]](#footnote-2) defined by the mid-point of the highest scoring ACS again pulls out 38 tRNA balls. This time the 2-sided p-value is a mundane 0.5, that is *according to this model* the association between *S. cerevisiae* ARSs and tRNAs is *not* statistically significant.

In *K. lactis* we find using the intergenic region model that our sample of 145 ARS balls[[2]](#footnote-3) taken from 5165 intergenic balls, of which 131 are tRNA balls[[3]](#footnote-4), pulls out 22 tRNA balls. The 2-sided p-value is 1.2e-11, however, in contrast with *cerevisiae* the result is still significant when switching to the intergenic position model: our sample of 145 ARS balls taken from 3168363 intergenic balls, 145915 of which are tRNA balls, pulls out 22 tRNA balls yielding a still significant 2-sided p-value of 1.9e-6. Although not necessary for our analysis we complete the picture by noting that the average length of an ARS-bearing *lactis* intergenic region is 950bp whereas the average length of the ARS-less intergenic regions is 605bp. Similarly, the average length of a *lactis* intergenic region with / sans a tRNA is 1114 / 600 respectively.

**An ACS-centric predict-and-verify approach to identifying *S. cerevisiae* ARSs**

How much does the effectiveness of our iterative approach to discovering *lactis* ARSs owe to the unusually long 50bp ACS motif? How well can it predict *cerevisiae* ARSs where the ACS is significantly shorter (11bp, 17bp and 33bp have been mentioned [3])? In particular, how does it compare with Oriscan that is another computational approach to discovering *cerevisiae* ARSs [3]. The latter relies on a fairly complicated computational model, which in addition to a 17bp ACS motif looks at a much wider region around it. Our method on the other hand simply models the ACS but it does rely on the biological feedback to help it successively improve the ACS PWM.

To level the playing field between the two approaches we adopt the same training set of 26 *cerevisiae* ARSs that is used in the Oriscan paper[[4]](#footnote-5). We also use the same initial 17bp PWM that the authors compiled from all biologically verified ACS matches in this training set. Starting with this PWM and initial set of verified ARSs we apply our iterative predict-and-verify approach to locating other *cerevisiae* ARSs. As a surrogate for the biological verification process we rely on the fairly exhaustive database of *cerevisiae* ARSs, OriDB. Since OriDB has three categories of ARSs: confirmed, likely and dubious we need to decide how to interpret each.

If our predicted match falls within a confirmed ARS it is naturally counted as a correct prediction. Similarly, we consider likely ARSs as functional ones as well. This decision is supported by the fact that in a random screen of *cerevisiae* ARSs we isolated 34 distinct *cerevisiae* ARSs of which 8 coincided with likely OriDB ARSs (24 coincided with confirmed OriDB ARSs, one was chimeric and another a novel ARS, see **Dataset** S9 for the complete list). Similarly, when testing for ARS activity in sequences surrounding 16 high scoring ACS matches in the *cerevisiae* intergenic regions that excluded all confirmed ARSs we found that all 7 of the matches that coincided with likely OriDB ARS were functional (2 more turned out as likely corrections to the coordinates of ARS215 and ARS219, 2 others were brand new ARSs, while the remaining 5 were not functional see **Dataset** S8 for the complete list). While we expect some of the likely ARSs, particularly the ones that lack a high scoring ACS match, are in fact non-functional, we believe that considering *all* of them as functional does not give our approach any unfair advantage in the comparison below. Especially since low scoring ACS matches are unlikely to come up as candidates in our approach. In addition to OriDB's confirmed and likely ARSs we also consider as functional the 3 new ARSs we identify here that were mentioned above (see **Dataset** S11).

Dubious ARSs, on the other hand, were considered as non-functional. This was supported by the observation that none of our 34 randomly screened ARSs intersected a dubious ARS. Similarly, none of the 16 high scoring *cerevisiae* intergenic matches we probed coincided with a dubious ARS. This is in sharp contrast to the fact that dubious ARSs cover over 50% more of the *cerevisiae* genome than the combined coverage of the likely and confirmed ARSs. More generally, any match that did not coincide with the functional set defined above was considered non-functional.

When assessing the performance of such a sequence-based predictor/classifier as in our approach or Oriscan, one has to keep in mind the repetitive nature of some ARS families. For example, there is a group of well-known core-X ARSs that are located near both ends of each of the 16 chromosomes [4]. The existence of such, essentially identical, sequences that share their functionality as well as their score can heavily bias the perceived performance of a classifier. For example, Breier at al. report that Oriscan’s list of top 100 predictions includes 94 functional ARSs. However, a closer examination of that list of top 100 shows it is riddled with duplicates[[5]](#footnote-6). Indeed, to generate the list of the top 100 *distinct* predictions (see below) we needed to consider the top 125 predictions in Oriscan’s original list, in other words there are 25 “hitchhiking” sequences among Oriscan’s top 125 predictions. The number of functional ARSs in the revised top 100 list is down to 84 (compared with 94 when duplicates are not ignored).

Accordingly, our set of functional ARSs was revised to remove duplicates. Of the core-X ARSs, which include 14 confirmed as well as 14 likely ARSs, we only kept ARS400. Of another repetitive family of ARSs, which we identified[[6]](#footnote-7) as telomeric to the core-X ARS and which includes confirmed OriDB ARS200 and ARS1001 as well as likely OriDB ARSs VII-1090, XII-1, XII-7, XII-1071, XII-1, XIV-1 and XV-1091, we only kept ARS200. In addition we BLASTed our reduced set of functional ARSs against itself and removed one sequence from each pair for which at least 50% of one of the sequences is covered by an alignment of at least 98% identity. This resulted in the further removal of 7 duplicated sequences. Corresponding to these filtered duplicates we defined a set of “ignored regions” so that only one match of each repetitive class would be considered (see **Dataset** S10 for a complete list of excluded regions and duplicates).

This “distinct” list of functional ARSs was then further filtered to remove all ARSs that coincide with our Oriscan-inherited training set of 26 ARSs. This left us with 503 (presumed) functional ARSs: 284 confirmed OriDB ARSs[[7]](#footnote-8), 216 likely OriDB ARSs and our 3 novel ARSs. Notably, 486 of these ARSs intersect intergenic regions and again we only concentrate on predicting those below.

In general, our method is iterative and relies on the biological verification feedback. However, in this case we essentially have all the verification data in advance. Thus, to avoid gaining an unfair advantage it is important to establish simple and consistent rules for defining each step in our iterative process. Relying on our experience with *lactis* we set the size of each new batch of candidates to 20 (due to the discrete character of the SADMAMA score some batches are slightly larger than 20 as the choice is between a number strictly less than or strictly bigger than 20). The first batch is an exception as it is generated based on a PWM compiled from only 26 sequences. We therefore consider only 14 candidate sequences for the first batch amounting to roughly 50% of the initial data (again the choice is between adding 12 and 14 candidates so we choose the higher number). With the exception of the initial PWM that we took as Oriscan's original matrix, later matrices are generated by GIMSAN applied to the cumulative set of "verified" ARSs. We set the width consistently to 35. This can be justified by a visual inspection of several lengths (see the logo below) and is consistent with a few papers that noted the unusual conservation to the right of the 17bp ACS (on the T-rich strand) [3] [5] [6].

At the end of 5 iterations of our method its top 100 predictions (which include all predictions from earlier steps) include in addition to 91 functional ARSs, 7 sequences whose functionality is unknown (and therefore assumed negative), 1 Dubious and 1 sequence that coincides with a sequence verified to be non-functional by our experiments alluded to above (**Dataset** S5). According to our definition this gives 9% failure rate. Compare this with the top 100 predictions of Oriscan (top 125 of the original list after removing all duplicated sequences) which include in addition to 84 functional ARSs, 14 sequences whose functionality is unknown, and 2 Dubious ones for a 16% failure rate (**Dataset** S6). Thus, our iterative method, which relies only on predicting the ACS, represents over 44% improvement over Oriscan's top 100 predictions. Note that if instead of using our iterative approach we only use Oriscan's original PWM to predict new candidates the failure rate increases to 22/99: these top 99 predictions include 20 sequences whose functionality is unknown, 1 Dubious and 1 sequence which was verified to be non-functional. If we take the top 102 candidates suggested by Oriscan's original PWM then the failure rate goes up to 24/102 (again, we cannot define the top 100 candidates due to the discrete nature of SADMAMA's score).

We stopped the comparison with the top 100 predictions mainly since it is rather painful to manually translate Breier et al.'s coordinates (unspecified genome version) to our reference genome (see discussion in technical section below). Still, the advantage of the predict-and-verify approach is quite evident. Recall that Oriscan uses a lot more information in the sequence than just its ACS. This leads us to believe that endowing our predict-and-verify approach with a more sophisticated prediction step, which will extract more from the sequence than merely its best ACS score, would further improve our results.


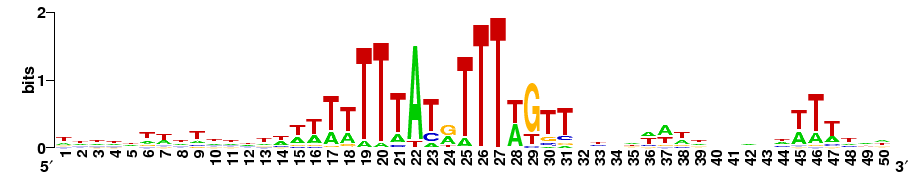


LOGO of a 50bp motif reported by GIMSAN applied to all verified cerevisiae ARSs. The 35bp extending between positions 13-47 suggests a width of 35 as a reasonable choice.

**Technical Details**

Finding the 26 training sequences of Breier et al. took some effort: only 25 sequences are annotated as such in their Additional Data File 1. On the other hand, their Additional File 8 that also contains information about the training set lists 3 additional sequences as being part of the training set. Two of those additional sequences are unnamed and are specified only by their coordinates while the third is given the name rDNA ARS which coincides with OriDB's ARS1216.5. To keep the training set at 26 sequences, we therefore assumed the two other sequences, both on chromosome 15 (1053697 and 908311), were erroneously annotated as being part of the training set.

Unfortunately, we could not find which version of the *cerevisiae* genome is Breier et al.'s ranked list of Oriscan candidates referring to (their Additional Data File 4). To overcome this issue we blasted the sequences of their training set as well those of their experimentally supported predictions ranking 350 or better (their Additional Data File 8) against the October 2003 genome. We then systematically compared their coordinates with the corresponding ones from our reference genome. Chromosome 3 showed significant change in coordinates (over 1000bp toward its right end) which required us to “manually” match each of their adjusted top 100 predictions that fell on that chromosome. Chromosome 4 has a significantly less problematic shift that was no worse than 50bp while all other chromosomes exhibited negligible shifts of coordinates. We addressed this issue by allowing some slack when comparing Brier et al.'s coordinates against our functional ARS set. Finally, we mapped the coordinates of the training set sequences that lie on chromosome 3 to our Oct 2003 reference genome based on the ARS name and/or blasting its sequence.

All OriDB ARS coordinates and types were downloaded from <http://www.oridb.org/> v.1.1.1. The coordinates are in the October 2003 version of the *S. cerevisiae* genome that was downloaded from SGD.

Intergenic sequences were generated from the GenBank files of the October 2003 genome by filtering out all sequences with feature type 'gene', 'LTR', or 'gap'.

The gibbsMarkov part of GIMSAN was applied to the set of previously verified ARSs using a training file consisting of all *S. cerevisiae* intergenic regions with the parameters:

-gibbsamp -l 35 -p 0.05 -best_ent -em 0 -t 200 -L 200 -ds -markov 4

In addition to the PWM reported by GIMSAN, SADMAMA was given the same training file as GIMSAN, which was also used as the input target set for scanning for matches to the PWM. Other parameters were:

-printScoresGTT first_set -siteThresholdLearnedFrom 1e-4 both_strands nullTrainFile -tests -- -pwmPC 0.01 -m 4 both_strands -siteNullScore avg_strands

**Supplementary Datasets**

1. lac_iterative_list_5_iters.csv – List of 123 predicted KlACS compiled from 5 systematic iterations of predict-and-verify (the 5^th^ cycle consisted of 41 predictions). The ‘functional’ column refers to the ARS functionality of the region defined by the 50bp predicted ACS plus the flanking 200bp. We could not clone five of the predicted ARSs (no entry under the ‘functional’ column). The first of these in particular (3 - 16994) resisted multiple cloning attempts. Interestingly, it is very close to start of chromosome C and it joins other lines of evidence indicating some mismatch in the telomeric regions between our strain’s genome and the published one[[8]](#footnote-9). Of the 118 predicted ARSs that we could clone, 75 exhibited ARS activity while 43 did not. Another 4 IGLARS were predicted and verified using other, less productive predictions (see **Dataset S2**).
2. lac_complementary_candidate_list_rev.csv – List of additional 36 predicted KlACS using a multiple of other techniques to computationally predict ARSs such as, favoring predictions that fall in long ARS-less stretches, scoring matches by ignoring weak columns, using cross validation techniques and looking for "dimeric matches" (matches that have a flexible number of spacers). The yield here was much lower: 4 verified functional vs. 32 verified as non-functional.
3. refined_pwm – The 50bp-wide PWM derived from the list of 148 verified ARSs with the exception of two telomeric ARSs (E/5 – 2231306 and F/6 - 2599876) whose similarity to the telomeric ARS at the end of chromosome C would have biased the estimated PWM. Similarly, the KARS12 was left out due to the suspected dimeric character of its ACS match.
4. conservation_p_values_table.doc – Summary of some of the ARS positional conservation analysis.
5. cer_iterative_list_exclusive.csv – List of 100 top ARS predictions compiled from 5 iterations of predict-and-verify starting with Breier et al.’s training set of 26 sequences and PWM (see details above). The last 5 columns are taken from OriDB based on coordinate matching. The 4 exceptions to the last statement are when under ‘ars_type’ are either ‘no’ (meaning this ARS was verified as non-functional) or ‘predicted-yes’ (meaning this ARS was verified to be functional). Note that all 4 ‘predicted-yes’ ARS are known likely OriDB ARSs only our versions here are significantly shorter (see also **Dataset** S8).
6. Breier_top_125_100.csv – List of top 100 distinct ARS predictions compiled from Breier et al.’s original list of top 125 candidates. In addition to removing ARSs belonging to the excluded regions (see above) we also noted that 6 candidate ARSs on the original appear twice: ARS1424 (42,104), ARS1109 (4,23), ARS1621 (20,91), ARS1628 (66,73), ARS702 (25,100) and (81,105) that are 24bp apart. There are seemingly 15 candidate ARSs whose functionality is unknown, however a closer look reveals that (117) Ch. 3, 271748 is probably a shifted coordinate version of ARS316 and should be counted as a correct prediction. Again, (102) is in fact a likely OriDB ARS. This leaves us with 14 predictions whose functionality is unknown and 2 dubious for a total of 16 failures.
7. cer_clean_verified_and_likely_IG_ARSs_ACS35.csv – List of 344 verified and high scoring likely *S. cerevisiae* intergenic ARSs. The ACS coordinates, strand and score refer to the predicted best match to a 35bp long ACS PWM as explained in the text. An ARS is classified as intergenic based on the center position of this match. ARSs with type “predicted” belong the list of ARSs we predicted based on their high scoring ACS match (**Dataset** S8) whereas type “seed_Ivan” refers to ARSs we randomly isolated in a screen (**Dataset** S9).
8. cer_novel_predictions_of_IG_ARSs_verified_col_shuffled.csv – Verification of 16 top high scoring ACS matches in the *S. cerevisiae* intergenic regions that excluded all confirmed ARSs. The last 4 columns are taken from OriDB based on matching coordinates. The ‘functional’ column refers to the ARS functionality of the 33bp predicted ACS taken with the flanking 200bp on each side. Of note: all 7 of predicted ARSs that coincide with likely OriDB ARS are functional, 2 more (Ch. 2 417582 & 591418) are most likely corrections to the respective coordinates of ARS215 and ARS219, 2 others (Ch.5 192693 & Ch.8 94959) are brand new ARSs we report here, while the remaining 5 are not functional. All ACS scores were based on a preliminary, width 33, PWM that was generated by GIMSAN from the list of confirmed OriDB ARSs sans all but one core X ARSs. This PWM was then further manually modified to remove all "non-interesting" columns and was used to scan the *S. cerevisiae* intergenic regions using a modified version of SADMAMA that allows gapped positions in the motif.
9. cer_seed_ARSs_intersection_with_oridb_no_dups.csv – Table of 34 distinct ARSs isolated in a random screen of *S. cerevisiae* ARSs. Columns 2-4 are derived from blasting the ARS sequence against the *S. cerevisiae* genome (October 2003). The last 4 columns are taken from OriDB based on matching coordinates. Of note: 8 of the screened ARSs coincide with likely OriDB ARSs, 24 coincide with confirmed OriDB ARSs, one is chimeric (303-100-1) and another (303-7-1), a novel ARS we report here.
10. cer_excluded_regions_intersect_oridb.csv – List of 54 “excluded” regions that were avoided in the prediction process. These consist of two large families of nearly identical ARSs: subtelomeric X-ARS and an ARS family which is telomeric to it (see text above), as well as a few other pairs of nearly identical ARSs of which one ARS from each pair was excluded (see ‘reason’ column). The last 5 columns are taken from OriDB based on matching coordinates.
11. new_cer_ARSs.tsv – Newly identified and better localized *S. cerevisiae* ARSs. The first ARS was identified through a random screen while the other nine through high scoring ACS matches followed by verification of the flanking segments. Of these nine, two are completely new while seven other offer verification and localized versions of more loosely defined likely ARSs. Note that 6 additional likely OriDB ARSs were verified in our random screen but they are not as compact as the predicted ones included here (**Dataset** S9).

**References**

1. Gordon JL, Byrne KP, Wolfe KH (2009) Additions, losses, and rearrangements on the evolutionary route from a reconstructed ancestor to the modern Saccharomyces cerevisiae genome. PLoS Genet 5: e1000485.

2. Wyrick JJ, Aparicio JG, Chen T, Barnett JD, Jennings EG, et al. (2001) Genome-wide distribution of ORC and MCM proteins in S. cerevisiae: high-resolution mapping of replication origins. Science 294: 2357-2360.

3. Breier AM, Chatterji S, Cozzarelli NR (2004) Prediction of Saccharomyces cerevisiae replication origins. Genome Biol 5: R22.

4. Louis EJ (1995) The chromosome ends of Saccharomyces cerevisiae. Yeast 11: 1553-1573.

5. Xu W, Aparicio JG, Aparicio OM, Tavare S (2006) Genome-wide mapping of ORC and Mcm2p binding sites on tiling arrays and identification of essential ARS consensus sequences in S. cerevisiae. BMC Genomics 7: 276.

6. Chang F, Theis JF, Miller J, Nieduszynski CA, Newlon CS, et al. (2008) Analysis of chromosome III replicators reveals an unusual structure for the ARS318 silencer origin and a conserved WTW sequence within the origin recognition complex binding site. Mol Cell Biol 28: 5071-5081.

1. There are 4 more balls in this model as a few ARSs share the same intergenic space. [↑](#footnote-ref-2)
2. ARS location was assigned by the mid-point of the best scoring lacACS match. [↑](#footnote-ref-3)
3. We used the tRNA annotation in the previously mentioned *lactis* genbank files. [↑](#footnote-ref-4)
4. Technically this turned out more challenging than it sounds: see the discussion under “Technical Details” below. [↑](#footnote-ref-5)
5. As well as multiple matches in the same ARS which they count separately [↑](#footnote-ref-6)
6. using SADMAMA [↑](#footnote-ref-7)
7. ARS215 and ARS219 were substituted according to the coordinates specified in **Dataset** S8 [↑](#footnote-ref-8)
8. In addition to several telomeric candidates that we failed to clone, our initial screen pulled *multiple copies* of ARSs that were clearly telomeric yet they did not perfectly match any of the telomeric sections of the published genome (nor anywhere else). [↑](#footnote-ref-9)
